# Genome–Scale Metabolic Networks Shed Light on the Carotenoid Biosynthesis Pathway in the Brown Algae *Saccharina japonica* and *Cladosiphon okamuranus*

**DOI:** 10.3390/antiox8110564

**Published:** 2019-11-16

**Authors:** Delphine Nègre, Méziane Aite, Arnaud Belcour, Clémence Frioux, Loraine Brillet-Guéguen, Xi Liu, Philippe Bordron, Olivier Godfroy, Agnieszka P. Lipinska, Catherine Leblanc, Anne Siegel, Simon M. Dittami, Erwan Corre, Gabriel V. Markov

**Affiliations:** 1 Sorbonne Université, CNRS, Integrative Biology of Marine Models (LBI2M), Station Biologique de Roscoff (SBR), 29680 Roscoff, France; 2 Sorbonne Université, CNRS, Plateforme ABiMS (FR2424), Station Biologique de Roscoff, 29680 Roscoff, France; 3 Groupe Mer, Molécules, Santé-EA 2160, UFR des Sciences Pharmaceutiques et Biologiques, Université de Nantes, 9, Rue Bias, 44035 Nantes, France; 4 Université de Rennes 1, Institute for Research in IT and Random Systems (IRISA), Equipe Dyliss, 35052 Rennes, France; 5 Quadram Institute, Colney Lane, Norwich NR4 7UQ, UK

**Keywords:** genome–scale metabolic networks (GSMNs), data integration, brown algae, oxygenated carotenoid biosynthesis, fucoxanthin, abscisic acid, *Saccharina japonica*, *Cladosiphon okamuranus*

## Abstract

Understanding growth mechanisms in brown algae is a current scientific and economic challenge that can benefit from the modeling of their metabolic networks. The sequencing of the genomes of *Saccharina japonica* and *Cladosiphon okamuranus* has provided the necessary data for the reconstruction of Genome–Scale Metabolic Networks (GSMNs). The same in silico method deployed for the GSMN reconstruction of *Ectocarpus siliculosus* to investigate the metabolic capabilities of these two algae, was used. Integrating metabolic profiling data from the literature, we provided functional GSMNs composed of an average of 2230 metabolites and 3370 reactions. Based on these GSMNs and previously published work, we propose a model for the biosynthetic pathways of the main carotenoids in these two algae. We highlight, on the one hand, the reactions and enzymes that have been preserved through evolution and, on the other hand, the specificities related to brown algae. Our data further indicate that, if abscisic acid is produced by *Saccharina japonica*, its biosynthesis pathway seems to be different in its final steps from that described in land plants. Thus, our work illustrates the potential of GSMNs reconstructions for formalizing hypotheses that can be further tested using targeted biochemical approaches.

## 1. Introduction

*Saccharina japonica* and *Cladosiphon okamuranus* are two brown algal species widely used in Asian aquaculture, known, respectively, as kombu and mozuku. *S. japonica* is the most important edible alga from an economic viewpoint. Its production was multiplied by 8 over the last 40 years: 0.8 million tons harvested in 1976 against 4.5 million tons in 2004 [1]. The annual production of *C. okamuranus* is estimated to be 20,000 tons [2,3]. Thus, studying the mechanisms of biomass production of those organisms through their Genome–Scale Metabolic Networks (GSMNs) may have a direct interest for algoculture [4]. Indeed, to produce molecules with high added value through biotechnological engineering, one first needs to understand their biosynthetic pathways. Brown algae produce specific carotenoids, some of them having potentially positive effects on human health, in particular, due to their antioxidant properties [5,6,7,8,9,10]. Some cleaved carotenoid derivatives are also signaling molecules and important phytohormones in land plants, like strigolactones or abscisic acid (ABA) [11,12,13].

Carotenoids are a ubiquitous class of molecules found in many organisms, such as plants, fungi, algae, and even metazoans. They are membrane–stabilizing hydrophobic molecules that also play a crucial role as photoprotective pigments in photosynthetic organisms [11,12,14,15]. Some carotenoids have been preserved through evolution, such as violaxanthin, which is detected in plants, green algae, and brown algae, among other organisms. This molecule is, on the one hand, the entry point for the biosynthesis of xanthophylls specific to brown algae, like fucoxanthin, which is the main marine carotenoid of this lineage [16,17,18] and, on the other hand, one of the precursors in the biosynthesis of ABA. Except for Laminariales [19], nothing is known about ABA in brown algae, including Ectocarpales like the model species *Ectocarpus siliculosus* or *C. okamuranus*. Given the potential importance of this signaling molecule in the biology of brown algae, one of our objectives when reconstructing the GSMNs of *S. japonica* and *C. okamuranus* was to clarify the possible contribution of land plant-like biosynthetic enzymes to the production of ABA in brown algae.

There have been extensive efforts to improve GSMN reconstructions from a computational perspective [20,21]. One current challenge is to integrate the knowledge of genome-based evidence and the knowledge coming from metabolome-based evidence, which is usually much more heterogeneous [22]. Following the protocol recommended in Palsson and Thiele [23] and in line with previous work on the model algae *E. siliculosus* [24], *Tisochrysis luteae* [21] and *Chondrus crispus* [25], the automated reconstruction methods and labor-intensive manual curation were combined to build a genome–scale metabolic model in the brown algae *S. japonica* and *C. okamuranus*, with a specific focus on the carotenoid biosynthesis pathway.

## 2. Materials and Methods

### 2.1. Data Sources and Cleaning

Genome and protein sequences of *S. japonica* and *C. okamuranus* are freely available, respectively, in references [26] and [3]. These algae are usually grown in non-axenic media, and the presence of contaminating sequences was demonstrated in the published genome of *S. japonica* [27]. Therefore, Taxoblast analysis (version 1.21beta) was carried out to discriminate prokaryote and eukaryote in order to filter out all prokaryotic sequences from the genome of *S. japonica*. Blast analyses were performed using diamond blastx (version 0.9.18) [28] against the nr database (downloaded on 13 August 2016) and the nodes.dmp (downloaded on 23 March 2018) from https://ftp.ncbi.nlm.nih.gov/pub/taxonomy/. The cleaned version of the *S. japonica* genome is available under the following link: http://abims.sb-roscoff.fr/resources/genomic_resources.

### 2.2. Reconstruction of Genome–Scale Metabolic Networks 

AuReMe (AUtomatic REconstruction of MEtabolic models—version 1.2.4) dedicated to “à la carte” reconstruction of GSMNs [21] was used to reconstruct the GSMNs of *S. japonica* and *C. okamuranus*. This workflow was installed locally from a docker image. As suggested for state-of-the-art GSMN reconstruction methods [23], this workflow allowed, through the encapsulation of several tools and the local installation of specialized tools in the docker container, to create a high-quality GSMNs based on genomic and metabolic profiling data. It combined 2 complementary approaches to extract information from the genome sequences. One results from the functional annotation of the genome (see Table S1 and Figure S1 of Supplementary Materials), and the other is derived from the comparison with GSMNs and protein sequences of organisms selected as templates. These intermediate networks were then merged, analyzed both qualitatively (topological analysis) and quantitatively (constraint-based analysis), refined by manual curation and enriched with metabolic profiling data extracted from the literature (see Table S2 and Table S3 of Supplementary Materials).

To reconstruct the intermediate annotation-based network, predicted coding regions in transcripts were functionally annotated using the Trinotate pipeline (version 3.0.1) [29]. An internal Trinotate script extracted the Gene Ontology Terms (GOT) [30,31]. The Kyoto Encyclopedia of Genes and Genomes identifiers (KEGG) [32] from the annotation file were used to retrieve the EC numbers associated with the genes. This step was performed via the KEGG–APi REST (REpresentational State Transfer) application programming interface (http://www.kegg.jp/kegg/rest/keggapi.html). The annotation concerning *S. japonica* was enriched by further analyses carried out with Kobas [33] and Blast2GO [34] with an e-value cutoff of 1e–05. A file created in the GenBank format, containing all the available data, was then generated using the following script: https://github.com/ArnaudBelcour/gbk_from_gff. This GenBank file was used as an input to the PathoLogic software from the Pathway Tools suite (version 20.5, default settings) [35]. The database containing the information from the annotation was then exported in attribute-value flat files, which were necessary for further analysis in the AuReMe workspace.

To reconstruct the intermediate orthology–supported network, templates from 3 model organisms were used: *Arabidopsis thaliana* [36], *E. siliculosus* [24], and *Nannochloropsis salina* [37]. Those templates were chosen according to the quality of their GSMNs or their phylogenetic proximity to the 2 studied algae. Orthology searches between the 2 studied algae, and these 3 templates were carried out using the OrthoMCL (version 1.4) [38] and Inparanoid (version 4.0) [39]. The results of the latter were combined using the pantograph (version 0.2) [37]. Since the *S. japonica* GSMN was the first to be generated, it was added to the 3 previous templates during the reconstruction of the *C. okamuranus* GSMN. As the *A. thaliana* and *N. salina* data refer to the KEGG [32] and BIGG [40] databases, respectively, a mapping operation, intrinsic to AuReMe, against the MetaCyc database (version 20.5) [41,42,43] was performed to standardize the identifiers with the MetaNetX dictionary [44].

Once the networks resulting from the annotation-based and orthology-based approaches were merged, an automatic gap-filling step was conducted using Meneco (version 1.5.0) [45]. This tool first tests the ability of the topological GSMNs to produce a set of metabolite targets defined by metabolite occurrences from the literature (Tables S2 and S3 of the Supplementary Materials) according to a Boolean abstraction of the metabolic network expansion [46]. We tested here whether or not metabolic paths existed between specific compounds known to be present in the studied algae. When one or more target(s) was (were) not reachable in the GSMNs, Meneco proposed a list of missing reactions to complete the GSMNs. For this qualitative analysis, it was necessary to provide 2 lists, one containing the target metabolites and the other containing a description of the culture medium, including the cofactors (seeds) essential for biochemical reactions (Table S4 of the Supplementary Materials).

One established criterion to consider the GSMN functional was the production of biomass with balanced growth. Flux Balance Analysis (FBA) is a widely used method to quantitatively model the behavior of the system under given conditions. This optimization problem was based on the principle of mass conservation and considered the steady state assumption (intracellular metabolites at equilibrium). Mathematically, this reaction was modeled by a linear function to be optimized. This point was tested using the quantitative FBA python scripts from the Padmet toolbox, based on the CobraPy package [47] and provided in AuReMe. To do this, the same production reactions, transport, and exchange of biomass used in the reconstruction of *E. siliculosus* GSMN [24] were added to our GSMNs (Table S5 of the Supplementary Materials). Biologically, the biomass reaction modeled the synthesis of essential amino acids, membrane lipids, and sugars by the organism. When the flux associated with a metabolite was nil, this implied the incompleteness of the GSMNs resulting either from missing reactions in the biosynthesis pathway or from an accumulation of one or more reaction products due to the absence of a degradation reaction for those metabolites. To overcome this issue, manual analysis was then carried out, either by adding outward transport reactions or by determining the reactions missing through the analysis of GSMNs of similar organisms. This manual analysis was guided by the predictions performed by the Fluto gap-filling tool [48].

The Venn diagrams presented in the results section illustrating the comparison between *S. japonica*, *C. okamuranus,* and *E. siliculosus* GSMNs were obtained using http://bioinformatics.psb.ugent.be/webtools/Venn/. The final versions of those GSMNs are available through their respective Wiki–websites: https://gem-aureme.genouest.org/sjapgem and https://gem-aureme.genouest.org/cokagem. They are designed to allow the visualization and specific search of the different components of a GSMN (pathways, metabolites, reactions, genes) [21]. The traceability of the reconstruction procedure was ensured by the display of the source(s) of each reaction: Annotations, orthology, and manual curation or gap-filling. These user-friendly wiki websites allow semantic searches according to the W3C standards, and they are designed to enable updates according to the expertise of the scientific community.

### 2.3. Exploration and Assessment of Carotenoid Biosynthesis Pathways in Brown Algae

The manual exploration of the carotenoid biosynthesis pathway (CAROTENOID–PWY) and the first cycle of xanthophylls (PWY–5945) was conducted starting from the pathways encoded in the MetaCyc database (version 22.6) [43] and completed by further literature searches. These 2 pathways encoded the activity of 10 enzymes whose protein sequences were selected, when identified, in *E. siliculosus* or, if not available, in *A. thaliana* (sometimes supplemented with other terrestrial plants). These protein sequences are accessible either via the Uniprot database (https://www.uniprot.org) or via the Online Resource for Community Annotation of Eukaryotes (ORCAE) website for *E. siliculosus* (https://bioinformatics.psb.ugent.be/orcae/overview/EctsiV2) [49]. Sequences homologous to these enzymes were then searched for in the proteomes of 3 green algae *Chlamydomonas reinhardtii*, *Volvox carteri*, *Ulva mutabilis*, 3 red algae *Chondrus crispus*, *Porphyra umbilicalis*, *Galdieria sulphuraria* and 6 stramenopiles *Nannochloropsis gaditana*, *N. salina*, *Phaeodactylum tricornutum*, *C. okamuranus*, *S. japonica*. With the exception of *Ulva mutabilis* and *E. siliculosus*, for which homology searches were carried out through the ORCAE portal [49], all proteomes are accessible in the Genome database (https://www.ncbi.nlm.nih.gov/genome) of the NCBI. All accession numbers used are available as Table S6 in Supplementary Materials. Proteome indexing was performed using formatdb (version 2.2.16), then homology searches were done with blastp (version 2.7.1+). Hits with an e-value of less than 1e–5 were selected for alignment after checking the organization of their protein domains (HmmerWeb [50] version 2.30.0—https://www.ebi.ac.uk/Tools/hmmer/search/phmmer). The sequence files previously obtained were aligned using the Muscle algorithm implemented in the Seaview software (version 4.4.2) [51]. The associated phylogenetic trees were generated in Seaview using maximum likelihood (PhyML) with the LG (Le et Gascuel) model, the BIONJ algorithm (BIO Neighbor–joining) for the starting tree, and 100 bootstrap replicates. The 5 trees presented in the supplementary data have been edited with Figtree (version 1.4.4). The modifications made to the GSMNs are detailed in Appendix A. Some of them are related to the second cycle of xanthophylls (PWY–7949), and fucoxanthin biosynthesis pathway (PWY–7950) encoded in MetaCyc.

## 3. Results

### 3.1. Genome–Scale Metabolic Network Reconstructions

Presented here are the functional GSMNs for *S. japonica* and *C. okamuranus*, both of which are of similar size. The *S. japonica* GSMN is composed of 3345 reactions and 2211 metabolites, and the *C. okamuranus* GSMN comprises of 3390 reactions, 2255 metabolites. Both GSMNs were expected to sustain biomass production since Flux Balance Analysis (FBA) analyses evidenced that their maximal growth rates were 3.67 mmol·gDW^−1^·h^−1^ and 3.56 mmol·gDW^−1^·h^−1^ (millimole per gram dry weight per hour), respectively. They constituted valuable tools for assessing and visualizing the currently available knowledge on the metabolism of these organisms.

**Figure 1:**
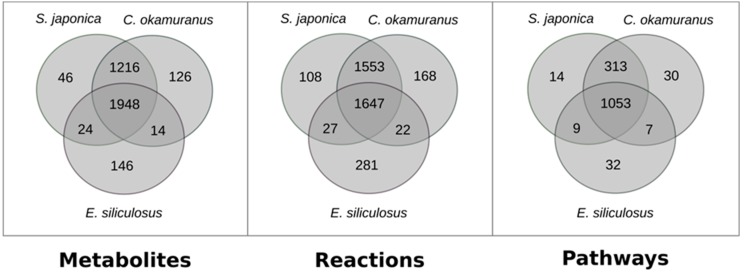
Comparison of the content of genome–scale metabolic networks of the three brown algae *S. japonica*, *C. okamuranus,* and *E. siliculosus.*

Both GSMNs were compared with the one from *E. siliculosus* [24], which was a brown algal model more closely related to *C. okamuranus* than to *S. japonica*. This was intended to test, at the global scale, if the differences between closely related species can be attributed to biological factors, or if they were merely due to technical differences in the reconstruction procedure. The results are shown in Figure 1.

**Figure 2:**
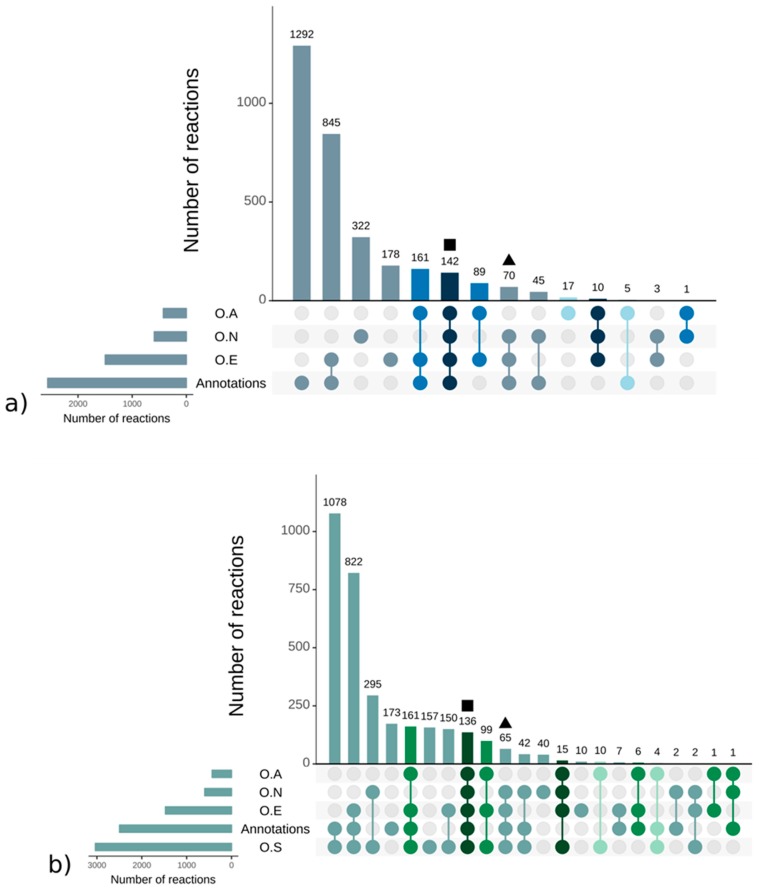
Sources of reactions in the *S. japonica* (**a**) and *C. okamuranus* (**b**) genome—scale metabolic networks. Reactions supported by orthology with *A. thaliana* (O.A), *E. siliculosus* (O.E), *N. salina* (O.N), and *S. japonica* (O.S). The different colors refer to the core reactions (blue and green gradient, from darkest to lightest). The square and triangle shapes are the examples corresponding to the mevalonate pathway (MVA), methylerythritol phosphate pathway (MEP), and the geranylgeranyl diphosphate pathways presented in the main text.

Strikingly, there were more reactions, metabolites, and pathways shared exclusively between *S. japonica* and *C. okamuranus* (1553 reactions) than exclusively between *E. siliculosus* and *C. okamuranus* (22 reactions), although the latter were phylogenetically closer. This was explained by examining the sources of reactions in the GSMNs (Figure 2). Of the reactions resulting from the annotations, 52% (*S. japonica*) and 44% (*C. okamuranus*) were not supported by the orthology with *E. siliculosus*. These differences were partly due to recent improvements in databases and annotation methods. The functional annotation of *E. siliculosus* that we used was done manually 10 years ago by an expert consortium [52], whereas we used Trinotate for *S. japonica* and *C. okamuranus* GSMNs. Therefore, the strong differences between *E. siliculosus* and the two other algae did not reflect biological differences, but different ways of annotation. One crucial point for the quality check was to build strong pathway-by-pathway expertise by scrutinizing them. AuReMe enabled the curation work to be stored and to reiterated on later versions of the GSMNs. To illustrate these points, intense manual curation centralized on the carotenoid biosynthesis pathway was performed.

### 3.2. Focused Exploration of GSMNs Regarding the Carotenoid Biosynthesis Pathway, Generalities, and Specificities

In order to facilitate the reading and understanding of the following sections, all the details related to the manual curation of GSMNs (names of enzymes or reactions, modifications of gene-reaction associations, adding or removing reactions, etc.) are available in Appendix A. Based on known pathways in terrestrial plants [53] and MetaCyc pathways (CAROTENOID-PWY), we conducted our exploration of the carotenoid pathway by starting with the transformation of geranylgeranyl diphosphate.

It should be noted, however, that this essential metabolic component was derived from isopentyl diphosphate (IPP). This fundamental and ubiquitous building block of the metabolism was itself obtained by the biosynthetic pathways methylerythritol phosphate pathway (MEP) and/or the mevalonate pathway (MVA) [54] (Figures S2 and S3 and Tables S7 and S8 of the Supplementary Materials). These pathways belonged to a set of reactions preserved through evolution that we will call core reactions. These reactions were easily and quickly identifiable within the GSMNs since they corresponded to those that have been inferred from the orthological search with a phylogenetically distant organism (here *A. thaliana*). In other words, these reactions were supported by all the components (orthology and annotations—square in Figure 2) that were used to build the GSMNs. Reactions associated with the enzyme, common to terrestrial plants and various algal phyla, which catalyzed the transformation of lycopene into β–carotene, also belonged to this set of core reactions.

**Figure 3:**
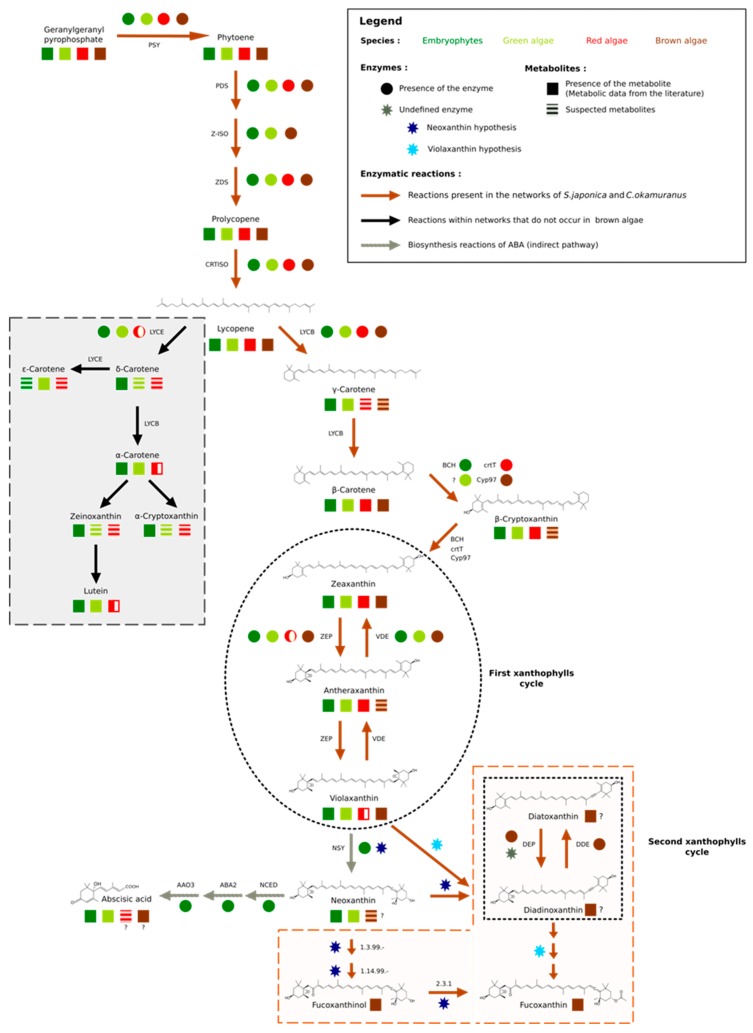
Carotenoid biosynthesis. The mechanisms of carotenoid synthesis seem to be common to all algae and terrestrial plants. The first connection point is made from lycopene. The synthesis of δ–carotene and its derivatives is absent in red microalgae and brown algae (grey insert), while the synthesis of β–carotene is also common to all organisms. The second point of divergence appears during the first xanthophyll cycle (black circle) partially found in red algae. In terrestrial plants, violaxanthin is transformed into neoxanthin, the starting point for the synthesis of abscisic acid (ABA), among other metabolites. Nevertheless, if this step exists in various algal groups, it seems to be carried out by different enzymes. Finally, stramenopiles have a second xanthophyll cycle (black square), for which one of the supposed precursors is also neoxanthin and whose enzymes remain to be determined (brown insert). Since pathways have been hypothesized for diatoms, we extended this hypothesis to brown algae. The figure is based on our results and the following references [5,53,55,56,57,58,59,60,61,62].

From geranylgeranyl diphosphate, five conserved enzymes were involved in producing lycopene (Figure 3). The genes of these enzymes have been characterized in green, red, and brown algae [55]. Homology searches based on *E. siliculosus* proteins coupled with network annotations confirmed the accuracy of the enzymes associated with those reactions (triangle in Figure 2) in *S. japonica* and *C. okamuranus*.

Among the terrestrial plants, red, green, and brown algae, lycopene was the first point of connection in the synthesis of carotenoids, since it offered two possible routes: The one of α–carotene and the one of β–carotene. These pathways started respectively, with the action of lycopene ε–cyclase (LYCE) for α–carotene and lycopene β–cyclase (LYCB) for β–carotene [63]. β–carotene is a carotene present in the majority of photosynthetic organisms, while α–carotene seems to be absent in stramenopiles and red microalgae [56]. These results were confirmed manually by our homology search. This search also allowed us to identify a single sequence containing one lycopene cyclase domain (PF05834) within the proteomes of brown algae: The lycopene β–cyclase [55,56,64] (Figure S4 of the Supplementary Materials). Nevertheless, within the GSMNs of the two brown algae, all the reactions leading to the synthesis of α–carotene and its derivatives (the grey part of Figure 3) were wrongly predicted and supported by this lycopene β–cyclase. Manual curation, therefore, led us to correct and suppress the reactions associated with α–carotene synthesis within the GSMNs.

The topology of the GSMNs, and in particular the presence of gaps, also revealed the specificities of the lineages. For instance, within the initially reconstructed GSMNs, no reaction allowing β–carotene transformation into zeaxanthin was automatically inferred. However, we know that this transformation involved different enzymes in different lineages, and in stramenopile species. Recent studies have suggested that this modification was catalyzed by an enzyme belonging to the P450 monooxygenase family [58,64]. During a homology search within the proteomes of *S. japonica* and *C. okamuranus*, we found two CYP97 contiguous paralogs sequences that we associated with this transformation (Figure 3).

### 3.3. No Plant-Like Abscisic Acid Synthesis Pathway in Brown Algae

**Figure 4:**
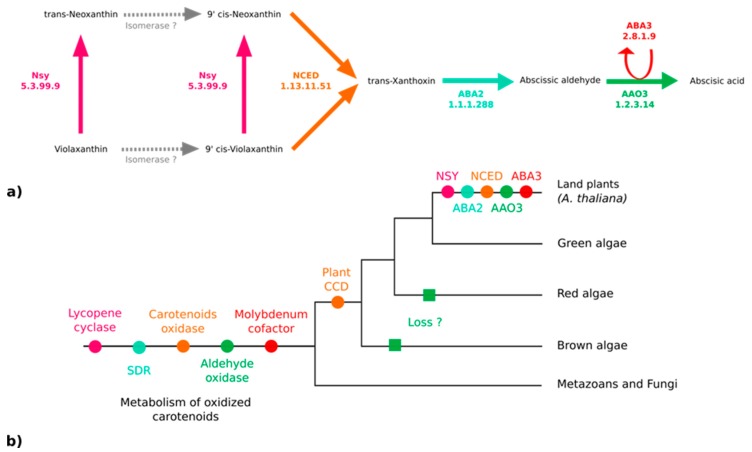
Enzymes of the abscisic acid biosynthesis pathway. (**a**) The ABA biosynthesis pathway in embryophytes (*A. thaliana*) [67]. (**b**) Phylogenetic tree representing the putative apparition and loss of the biosynthetic enzymes. NSY: Neoxanthin synthase; NCED: Abscisic–aldehyde oxidase; ABA2: Xanthoxin dehydrogenase; ABA3: Molybdenum cofactor sulfurase; AAO3: Abscisic–aldehyde oxidase; SDR: Short-chain alcohol dehydrogenase/reductase; CCD: Carotenoid cleavage dioxygenase.

Because ABA has been reported in various kelp species [19] and associated with biological effects on kelp growth and maturation [65,66], we thought it would be important to clarify if both *S. japonica* and *C. okamuranus* can produce it. The enzymes responsible for the synthesis of ABA from violaxanthin have been identified and characterized in embryophytes [67]. According to the MetaCyc pathway PWY–695, five enzymes are essential for the biosynthesis of ABA [67,68,69,70] (Figure 4a). We searched for orthologs of these five proteins in the brown algal genomes, but only paralogs were found (Figure 4b). All of them are members of large multigenic families whose conserved domains are general and, therefore, prevent the inference of their precise catalytic activities (Figure 4b and Figures S4–S7, and Table S9 of the Supplementary Materials).

We also examined two reactions involved in the regulation of ABA availability through its conjugation with glucose by an ABA glucosyltransferase (AOG — RXN–8155). Glycosylated ABA is inactive and can be stored for regulatory purposes. The activation of glycosylated ABA pools was performed by glucosidase (BG1 or BG2 — RXN–11469) [71,72]. However, a homology search carried out on these enzymes in predicted brown algal proteomes did not reveal any homologous sequences either.

### 3.4. Extending the Fucoxanthin Biosynthesis Models from Diatoms to Brown Algae

With the exception of the interconversion of zeaxanthin, antheraxanthin, and violaxanthin, violaxanthin was not consumed and corresponds at the GSMN level, to a dead end [73]. This, in silico dead end does not represent a biological reality since we know that this molecule is the entry point for the biosynthesis of fucoxanthin, but also of diatoxanthin and diadinoxanthin, two xanthophylls strongly suspected of being present in brown algae [56,59]. The reconstruction of the GSMNs was based, among other things, on the functional annotation of their genome, but on average, 35% of the predicted coding sequences in the two algae were devoid of annotations (See Figure S1 of Supplementary Materials). Among the sequences without any type of annotation, 28% (1984 *S. japonica* sequences) and 22% (1015 *C. okamuranus* sequences) can be considered as taxonomically restricted or orphan genes (sequences from one species not homologous to the other species). They represent on average, 11% and 7% of all predicted coding sequences. The other sequences without annotation 5088 and 3640 sequences in *S. japonica* and *C. okamuranus,* respectively, represent conserved proteins of unknown function. These unannotated sequences constitute a protein reservoir with unknown biochemical functions, probably including these sought enzymes.

The number and nature of cycling xanthophylls, metabolites involved in photoprotection and which depend on light conditions, differ among the algal lineages (black insert Figure 3) [59,60,74,75]. Based on previous studies, two distinct cycles have been described in stramenopiles: The zeaxanthin to violaxanthin interconversion cycle [74,75] and the diatoxanthin to diadinoxanthin interconversion cycle [59]. The enzymes involved in the first cycle are zeaxanthin epoxidase (ZEP) and violaxanthin de-epoxidase (VDE). The sequences corresponding to those involved in the second cycle, diadinoxanthin de-epoxidase (DDE) and diatoxanthin–epoxidase (DEP), have not yet been identified. However, it would appear that these enzymes are very close to ZEP and VDE since they catalyze the same type of reaction (opening or epoxide formation on the cyclic ends of molecules) [5,18,60]. Indeed, it has previously been suggested that one copy of the VDE gene, identified as VDL, could correspond to the DDE enzyme [5,60]. Within the various algal proteomes queried, we found only one sequence associated with the ZEP and, as expected, three known out–paralogues of VDE: VDE, VDR-related, and VDL-like (Figure S8 of the Supplementary Materials). Consistent with the absence of a xanthophyll cycle, no copy of the VDE gene was found in red algal proteomes, and the presence of a VDL appeared to be a specificity of stramenopiles [5,61]. We decided to associate this sequence with the reaction catalyzed by the DDE within our reconstructions

Finally, a hypothetical pathway leading to the production of fucoxanthin has been proposed in *P. tricornutum* (diatoms), but the enzymes are not yet known biochemically [60]. Nevertheless, two production hypotheses exist: Either through neoxanthin, whose presence is discussed in the literature [5,56,60,76] or directly through violaxanthin [18,60,61]. However, the investigation of the proteomes of the two brown algae has shown that the conversion of violaxanthin to neoxanthin, if it takes place, cannot be performed by neoxanthin synthase (NSY), as in terrestrial plants [53,77]. Whatever the hypothesis considered, in view of molecular structures, these biosynthetic steps require the intervention of at least three enzymes. It is likely that the latter, if they exist, are in the set of non-annotated sequences. Awaiting biological confirmation, we propose these two pathways in our reconstructions (brown insert in Figure 3).

## 4. Discussion

The use of automatic or semi-automatic, GSMN reconstruction workflows allows efficient and rapid modeling of GSMNs, even for emerging model organisms, as is the case for *S. japonica* and *C. okamuranus*. The theoretical biomass reaction we have tested here is mainly composed of the list of amino acids and some essential sugars. All these compounds were predicted to be producible, and our GSMNs are considered functional. In fine, for instance, adding compounds such as fucoxanthin, a molecule with pharmaceutical interest [17], could make it possible to understand and improve their production in the coming years

The reconstruction of the GSMNs is partially based on the assumption that two orthologous sequences, resulting from a speciation event, share the same function. Such reactions, called core reactions, reflect an ancient evolutionary origin and indicate which metabolites are preserved through the various kingdoms of life. For the most part, they correspond to metabolites historically qualified as primary (amino acids, essential sugars, ATP, ADP) but also ubiquitous secondary metabolites such as lycopene. The preliminary steps to the IPP synthesis are highlighted by the orthology component of the reconstruction, suggesting and confirming their ancestral origin. In general, all the reactions present within the GSMNs that are supported by orthology with *A. thaliana* illustrate this phenomenon. However, the list proposed based on this criterion is not exhaustive since, on the one hand, the *A. thaliana* GSMN preferentially targets “primary metabolites” and, on the other hand, some reactions may have been lost during the mapping steps between the various databases. This point is illustrated by the reactions allowing the synthesis of lycopene from geranylgeranyl diphosphate (triangle in Figure 2). They belong to the core reaction set (similar enzymes in plants and various algal lineages) but are not supported by the *A. thaliana* component. In contrast, reactions supported only by annotation may indicate a specificity of the species or lineage.

*A contrario*, the presence of gaps, and *a fortiori* dead ends within GSMNs has a high predictive power by informing either on the computational limit and/or biological specificity related to one species. From a purely bioinformatic point of view, the presence of gaps is directly related to the quality and quantity of data present in the databases. Apart from encoding issues of some metabolites, there is currently little *Phaeophyceae* referenced data. As such, a gap in the GSMNs may be a direct reflection of the biological specificities of the studied lineages and open up to new questions and biological investigations (i.e., identification of fucoxanthin biosynthesis enzymes). The first source of answers is probably to be found in the set of unannotated sequences, which are not taken into account in the reconstruction of the GSMNs. Indeed, the reconstruction is based on the functional annotation of coding sequences. Among all sequences, about 35% of them do not have any annotation and are of sufficient size to encode functional enzymes (average of 525 amino acids — see Figure S9 of the Supplementary Materials). There is no doubt that these sequences constitute a reservoir/pool of candidates for the shadows that persist within the current knowledge of brown algae. Moreover, diversifying the tools used during the annotation step could be considered in order to compensate slightly for computational artifacts listed above. Indeed, using Kobas and Blast2GO on *S. japonica* data allow finding 3.2% additional GO terms and 6.0% Kegg identifiers, which are necessary to track EC numbers (Figure S10 of the Supplementary Materials) Among the unannotated sequences, some may be candidates to be assigned to a reaction inferred by gap-filling without any known associated enzymes. Correlating gene expression data, targeted chemical profiling can help to narrow down candidates for full biochemical characterization, as already done in some model bacteria [78].

The GSMNs have been tested and enriched with targeted metabolic profiling data from the literature. Few or none of these targets were reachable before the gap-filling stage. The effectiveness of this step, more than two-thirds of the targets are now achievable, allowing us to propose a better topology of the GSMNs that can mimic the biological behavior of the brown algae (see Appendix A). Nevertheless, despite the presence of a positive flux under constraint-based modeling, some of the target metabolites are topologically not structurally producible in the GSMNs. These problems of unproducible compounds within bacterial and eukaryotic GSMNs are known [22,25]. The first intuitive constatation is that if the targets are not found during the topological analysis, this implies that at least some genomic data (e.g., an enzyme) is missing and that there is a need for further development of functional approaches. However, among the 12 and 7 unreachable targets—in *S. japonica* and *C. okamuranus* GSMNs, respectively—we found mainly fatty acids, which are rarely found in their free forms. By focusing on conjugated forms, particularly with acetyl–CoA, we realize that some of these compounds are present in the GSMNs thanks to the addition of gap-filling reactions. To summarize, the non-producibility of these targets can also come from the encoding choice of the referenced metabolites, and in this case, the GSMNs already reflect the biochemical reality of fatty acid metabolism. An additional category of metabolites is those that could even not be incorporated as targets for gap filling because they are not yet connected to biochemical reaction models and thus not incorporated in the Metacyc database. For such molecules, specific tools have been developed to infer new reaction models using detailed comparisons of already known reactions and molecules substructures or to infer the precise structures of the unknown intermediates leading to known metabolites [25].

The curation of the carotenoid pathway has pointed out one of the limits of automatic GSMN reconstructions with the proposal of genes and reactions leading to the synthesis of α–carotene and its derivatives. This point highlights the need for manual curation steps since, in this specific case, only the user’s expertise could make it possible not to infer these reactions. Indeed, the in silico proposal of these pathways is not aberrant since the enzyme that catalyzes the transformation of lycopene into α–carotene, LYCE, is very similar to LYCB, which transforms the same substrate into β–carotene [57,77,79]. The approaches used here are based on the EC numbers of enzymes, and in this case, the proximity of their EC numbers (EC 5.5.1.18 and EC 5.5.1.19 respectively) explains why these undesired reactions were added and associated with the LYCB sequence.

In any case, as we have seen, these two in silico reconstructions of GSMNs provide a satisfactory model of the carotenoid biosynthesis pathway. We highlighted, on the one hand, the common skeleton (reactions and enzymes) that have been preserved through evolution and, on the other hand, the specificities related to brown algae. One of them corresponds to the presence of a second cycle of xanthophylls, and even if we are not able to propose a synthesis pathway with certainty, we propose a candidate for one of the two enzymes involved in the interconversion of diatoxanthin and diadinoxanthin. Another major point is the production of fucoxanthin. Here, we extend the biosynthetic hypotheses that were previously formulated in diatoms to brown algae.

On the contrary, there is not enough knowledge yet to identify the ABA synthesis pathway in filamentous brown algae from the kelp data for a number of reasons. In the European kelp species *Laminaria hyperborea*, *Laminaria digitate,* and *Saccharina latissima,* the presence of ABA in sporophytic tissues were reported about 20 years ago [19]. The characterization of this phytohormone has been performed by GC–MS (gas chromatography coupled with mass spectrometry). It has been reported that ABA concentration varies according to the seasons, with a maximum peak around November, and that a negative correlation exists between the increase in ABA concentration and vegetative growth of sporophytes [19]. Later, the presence of ABA was also detected in *S. japonica* by LC–MS/MS analysis (liquid chromatography coupled with two tandem mass spectrometers) [80]. In addition, it has been shown that the application of exogenous ABA may inhibit sporophyte growth and induce the accelerated formation of reproductive tissues called *sori* [65]. ABA has also been proposed to play a role in the control of elicitor-induced oxidative bursts [66].

Two pathways for the biosynthesis of ABA are known in the literature: The direct pathway and the indirect pathway. Chemically, two other pathways related to the regulation of ABA production have been proposed, but they are not supported by characterized enzymes thus far. ABA could be obtained by transformation of acid xanthoxin resulting from the oxidation of xanthoxin or by abscisic alcohol resulting from the oxidation of abscisic aldehyde [69]. In fungi, ABA is produced by the direct pathway within the cytoplasm from farnesyl diphosphate with ionylidene derivatives. In terrestrial plants, the existence of a pathway derived from farnesyl diphosphate is assumed but has not yet been identified [67]. The indirect pathway, the one explored here (see Figure 4a), is carried out in the plastids of the photosynthetic organisms following carotenoid cleavage [72,77,81]. Violaxanthin is transformed into neoxanthin by an intramolecular oxidoreductase, neoxanthin synthase (NSY). A series of structural modifications likely carried out by isomerases produces the 9-cis forms of the epoxycarotenoids (C40) violaxanthin or neoxanthin (main epoxycarotenoid) [67]. These two molecules undergo oxidative cleavage by a 9-cis-epoxycarotenoid dioxygenase belonging to the NCED family to form xanthoxin (C15). After the export of xanthoxin to the cytosol, an enzyme (ABA2) of the SDR family (short-chain alcohol dehydrogenase/reductase) oxidises this molecule. The opening of the epoxy forms the abscisic aldehyde. Finally, an abscisic aldehyde oxidase acid (AAO3) oxidises the abscisic aldehyde to ABA. This transformation is only achievable in the presence of a molybdenum cofactor sulfurase (MoCo - ABA3) that catalyzes the sulfarylation of a dioxo form of MoCo into a mono-oxo sulfide form necessary for the activation of abscisic aldehyde oxidase acid [82,83]. Homology searches did not allow us to find any orthologs of the corresponding genes in brown algae (see Figure 4b, Figures S4–S7, and Table S9 of Supplementary Materials). Thus, the paralogue sequences identified during the homology searches may be involved in the metabolism of carotenoids, but not specifically in the metabolism of plant-like intermediates in the ABA synthesis pathway from violaxanthin and this pathway, therefore, probably emerged in terrestrial plants.

To conclude, if brown algae are able to synthesize ABA, the corresponding pathway is either unknown at the moment or it could be close to that of fungi. Collecting metabolic data about biosynthetic intermediates would be key to discriminate between both hypotheses. Another possibility is that the metabolite reported as ABA is another structurally close oxidized carotenoid, like β–ionone, which could have an equivalent role [84]. Aside from ABA, there is a huge diversity of oxidized carotenoids involved in signaling processes [85], which gives ample room for possible structural variation in signaling molecules across lineages. Facing such diversity, integrative approaches through genome–scale metabolic models should be helpful tools to prioritize further experimental efforts, in a context where the discovery of drugs coming from natural products is experiencing a revival, fuelled by an increasing integration with genomics data [86].

## Figures and Tables

**Figure A1:**
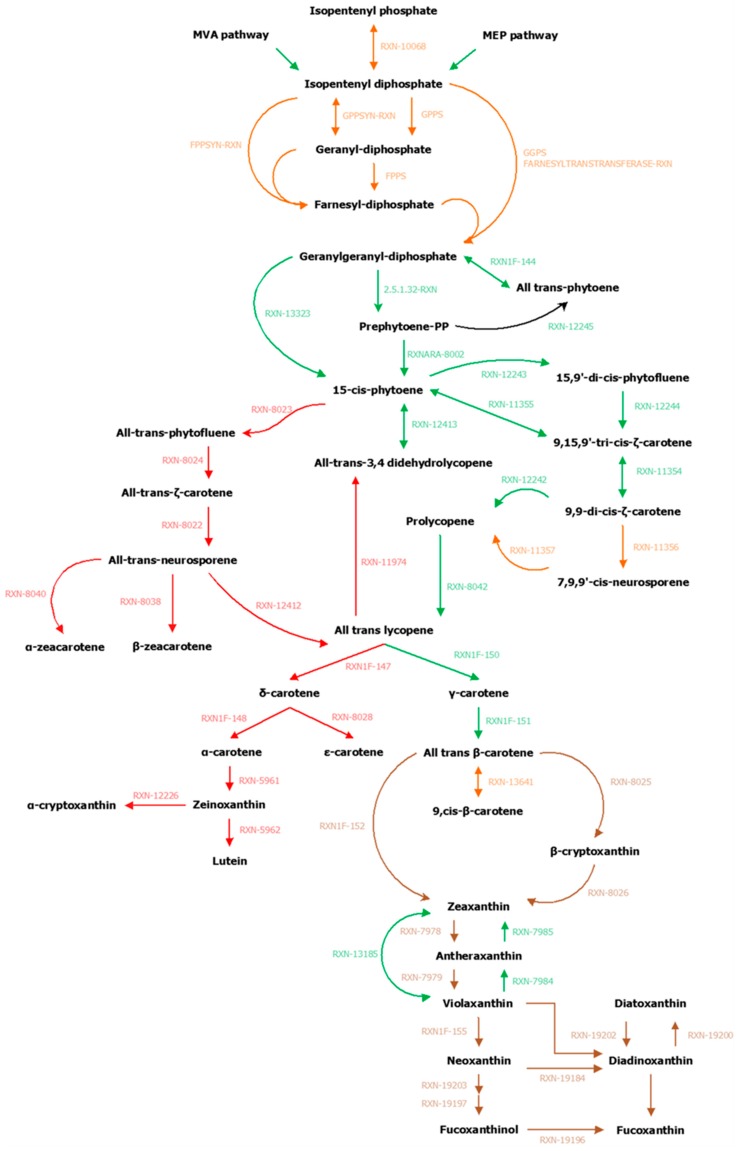
Reconstruction and manual curation of the genome–scale metabolic networks of *S. japonica* and *C. okamuranus.* In green, the reactions present initially in the GSMNs, in red those that were deleted, in brown those added, and in orange those to be checked.

## Supplementary Materials

The following are available online at https://www.mdpi.com/2076-3921/8/11/564/s1. **Table S1:** Data from the annotation of algal protein–coding genes, **Figure S1:** Source of the *S. japonica* (**a**) and *C. okamuranus* (**b**) annotations, **Table S2:** Targets for *S. japonica* from metabolic data in the literature [19,87,88,89,90,91,92,93,94,95], **Table S3:** Targets for *C. okamuranus* from metabolic data in the literature [2,96,97,98,99], **Table S4:** List of cofactors (seeds) added to the *S. japonica* and *C. okamuranus* GSMNs, **Table S5:** List of biomass reactions added to the *S. japonica* and *C. okamuranus* GSMNs [24], **Table S6:** Accession numbers of enzymes and protein sequences used, **Figure S2:** Pathways of methylerythritol phosphate (MEP) within the *S. japonica* and *C. okamuranus* GSMNs [43,54], **Table S7:** List of reactions and genes associated with the MEP pathways [43,54], **Figure S3:** Pathways of mevalonate (MVA) within the *S. japonica* and *C. okamuranus* GSMNs [43,54], **Table S8:** List of reactions and genes associated with the MVA pathways [43,54], **Figure S4:** Maximum likelihood tree of the lycopene cyclase family [57,77,100], **Figure S5:** Maximum likelihood tree of the NCED family [12,14,101,102,103,104,105], **Figure S6:** Maximum likelihood tree of the xanthine oxidase family [106,107], **Table S9:** Type of SDR found in the genomes of *S. japonica* and *C. okamuranus* [108], **Figure S7:** Maximum likelihood tree of the Mocos family [109,110,111,112,113], **Figure S8:** Maximum likelihood tree of the VDE [5,18,60,61], **Figure S9:** Amino acid length of unannotated *S. japonica* and *C. okamuranus* coding sequences, **Figure S10:** Enrichment of the annotation in GO terms and KEGG identifiers: data fusion. The wiki–website is available under the following links: https://gem-aureme.genouest.org/sjapgem/index.php/Main_Page and https://gem-aureme.genouest.org/cokagem/index.php/Main_Page.

## Appendix A

### A.1. Qualitative Analysis of Genome–Scale Metabolic Networks and Gap-Filling

Lists of 61 and 29 metabolites were compiled for *S. japonica* and *C. okamuranus* (Tables S1 and S2 of Supplementary Materials) and used to test the topology of the GSMNs. According to topological criteria modeling the Boolean behavior of the system dynamics and before the gap-filling step conducted by Meneco, five targets (L–α–alanine, mannose–1–phosphate, GDP–mannose, D–mannitol 1–phosphate, GDP–mannuronate) were reachable in the *S. japonica* GSMN. The topological gap-filling allowed reaching 45 and 22 additional metabolites by adding 90 and 67 gap-filling reactions, respectively, in *S. japonica* and *C. okamuranus* GSMNs, including 46 common reactions to both algae. The targets that could not be reached were mainly fatty acids. From a dynamic point of view, the presence of a target was then verified and supported by the possibility of producing a flux. This means that all import, export, and production reactions of these compounds were present in the GSMNs. In our analyses, all targets had a positive flux.

### A.2. Manual Curation Made to the S. japonica and C. okamuranus Genome–Scale Metabolic Networks

All modifications are reported in Table A1 and Figure A1.

Carotenoids (xanthophylls and carotenes) are synthesized from geranylgeranyl diphosphate, which was transformed into phytoene and then into lycopene under the action of five enzymes, three of them being common to all photosynthetic organisms: Phytoene synthase (PSY — EC 2.5.1.32), phytoene desaturase (PDS — EC 1.3.5.5) and ζ–carotene desaturase (ZDS — EC 1.3.5.6). As the genes of these enzymes are known in algae [55], a homology search based on *E. siliculosus* proteins coupled with annotations from the GSMNs have made it possible to identify one candidate for PSY (SJ02885 and g11610), one candidate for PDS (SJ08891 and g10852), and one to two candidates for ZDS (SJ05680,SJ05681 and g16199) in *S. japonica* and *C. okamuranus*, respectively.

The two other steps in the synthesis of all-trans lycopene were either catalyzed by ζ–carotene isomerase (Z–iso — EC 5.2.1.12) and prolycopene isomerase (crtISO — EC 5.2.1.13) or compensated by photoisomerization [53,62,102]. Based on the results published in reference [55], annotations, and homology searches, it appeared that one copy of the Z–iso gene (SJ04715 and g9721) was found in the brown algae. However, two and three copies of crtISO were found in *S. japonica* and *C. okamuranus*, respectively. According to the evolutionary history of these sequences, the most likely hypothesis would be the loss of the copy annotated as amine oxidase in *S. japonica* after a successive triple duplication [55]. Therefore, we propose two potential candidates for this gene for each of the algae, one of which is annotated with the expected EC number (SJ05083 and g8850) and the other without EC annotation (SJ22161 and g4521).

All-trans lycopene is the first carotenoid from which the biosynthesis pathway diverges since it allows forming both β–carotene and α–carotene. The cyclization of lycopene is ensured by a lycopene cyclase, which adds one β–ring (lycopene β–cyclase — EC 5.5.1.19) or one ε–ring (lycopene ε–cyclase — EC 5.5.1.18) at the extremity of the molecule [63]. The α–carotene, composed of a ε–ring and a β–ring is the precursor of lutein while the β–carotene, composed of two β–rings is the entry point for xanthophylls biosynthesis. The search for proteins homologous to lycopene β–cyclase (LYCB) and lycopene ε-cyclase (LYCE) of *A. thaliana* coupled with the check for the presence of the lycopene cyclase protein domain PF05834 made it possible to identify a lycopene cyclase sequence for each of the studied algae (SJ04962 and g10898). The reconstruction of the phylogenetic tree (see Figure S4 of the Supplementary Materials for more details) of the previously selected sequences allows linking these sequences to the lycopene β–cyclase, thus confirming the absence of α–carotene and its derivatives in these two brown algae [55,56,57]. However, all these reactions were inferred from the GSMNs and were supported, among other things, by the gene identified as LYCB (EC 5.5.1.19). This point, therefore, led us to remove from the GSMNs reactions associated with the synthesis of δ–carotene (RXN1F–147), α–carotene (RXN1F–148), ε–carotene (RXN–8028), zeinoxanthin (RXN–5961), α–cryptoxanthin (RXN–12226—only for *C. okamuranus* GSMNs) and lutein (RXN–8962).

The addition of a hydroxyl group at each end of the β–carotene forms zeaxanthin. Within the reconstructed *S. japonica* and *C. okamuranus* GSMNs, no reaction allows this modification. However, there are two reactions (RXN–8025 and RXN–8026) within the MetaCyc database that ensure this transformation using an intermediate, β–cryptoxanthin. The enzyme responsible for this transformation in algae is probably a monooxygenase belonging to the P450 family and more particularly of the CYP97B type [58,64]. In each of the genomes, two genes (SJ07227 and SJ07228 for *S. japonica*; g4983 and g4984 for *C. okamuranus*) were identified as such and one of them could ensure the hydroxylation of β–carotene to zeaxanthin [58].

The first cycle of xanthophylls in brown algae involves two enzymes and allows, depending on light conditions, the interconversion of zeaxanthin to violaxanthin through antheraxanthin [59,74]. In poor light conditions, a zeaxanthin epoxidase (ZEP — EC 1.14.15.21) causes the opening of the cycles positioned at the ends of zeaxanthin and then antheraxanthin to form epoxides. Within the algal genomes, there is a copy of this enzyme (SJ19373 and g5910), but only one reaction, that corresponding to the transformation of antheraxanthin into violaxanthin (RXN–7979) appears within the GSMNs. As a result, the reaction allowing the transformation of zeaxanthin into antheraxanthin (RXN–7978) was added manually to the GSMNs. In excess light, violaxanthin de–epoxidase (VDE — EC 1.23.5.1) converts violaxanthin into antheraxanthin (RXN–7984), which is then further modified to create zeaxanthin (RXN–7985). Within the GSMNs obtained, there is also a reversible reaction (RXN–13185) allowing the direct conversion of violaxanthin to zeaxanthin. For each of the algae, three sequences are associated with these reactions and the analysis of their phylogeny makes it possible to identify the three known out–paralogs: VDE (SJ03764 and g11316), VDL (VDE–like SJ20456 and g16187), and VDR (VDE–related, SJ19927 and g4586). The second cycle of xanthophylls, known in diatoms [60,61], and suspected in brown algae [56,59] operates on the same principle as the first cycle. Thus, the reactions linked to the interconversion of diatoxanthin into diadinoxanthin (RXN–19200 and RXN–19202) were added to the GSMNs. VDL (SJ20456 and g16187) has been associated with the reaction that converts diadinoxanthin to diatoxanthin (RXN–19202) [5,61] (see Figure S8 of the Supplementary Materials for more details).

According to the two hypotheses reviewed in reference [18], we have added seven reactions related to the synthesis of fucoxanthin and diadinoxanthin. According to the violaxanthin hypothesis [59], we created two reactions to allow the transformation of violaxanthin into diadinoxanthin (opening of the epoxide and formation of a triple bond—probably related to the action of a single enzyme) and the transformation of diadinoxanthin into fucoxanthin (reactions probably carried out by three independent enzymes). According to the neoxanthin hypothesis, we have added the three reactions presented in reference [60] (RXN–19203, RXN–19197 and RXN–19196) and also chose to add the reaction related to the transformation of violaxanthin into neoxanthin (RXN1F–155), even if there is no NSY in the two brown algae. Finally, a reaction allowing the transformation of neoxanthin into diadinoxanthin having been proposed by reference [60], we also added it to the GSMNs.

**Table A1:** List of manual modifications made to the GSMNs of *S. japonica* and *C. okamuranus*.

Enzymes	ID Reactions (MetaCyc)	Publication	*S. japonica* (Associated Genes)	*C. okamuranus* (Associated Genes)
Carotenoid biosynthesis and first xanthophyll cycle, well–known reactions whose genes are characterized in brown algae				
PSY (phytoene synthase)	RXN–13323	[11,53,55,61,62,104]	SJ02885	g11610
	2.5.1.32–RXN			
	RXNARA–8002			
PDS (phytoene desaturase)	RXN–12243	[11,53,55,61,62,104]	SJ08891	g10852
	RXN–12244			
	RXN–11355			
Z–iso (ζ–carotene isomerase)	RXN–11354	[55,104]	SJ04715	g9721
ZDS (ζ–carotene desaturase)	RXN–12242	[11,53,55,61,62,104]	SJ05680 SJ05681	g16199
	RXN–11356			
	RXN–11357			
crtISO (prolycopene isomerase)	RXN–8042	[55,56,61,62,104]	SJ05083	g8850
LycB (lycopene β–cyclase)	RXN1F–150	[11,53,55,56,57,61,62,63,104]	SJ04962	g10898
	RXN1F–151			
Cyp97B	RXN–8025	[12,58,62,64,104]	SJ07227 SJ07228	g4983 g4984
	RXN–8026			
	RXN1F–152			
ZEP (zeaxanthin epoxidase)	RXN–7979	[11,18,53,55,56,62,74,104]	SJ19373	g5910
	RXN–7978			
VDE (violaxanthin de–epoxidase)	RXN–7984	[11,18,53,56]	SJ03764 SJ20456 VDL (VDE–like ~ DDE) SJ19927 VDR (VDE–related)	g11316 g16187 VDL (VDE–like ~ DDE) g4586 VDR (VDE–related)
	RXN–7985			
Reactions removed				
phytoene desaturase (fungi – al–1 or crtI)	RXN–12413		SJ18358 (ANNOTATION)	g18971.t1 (ORTHOLOGY)
	RXN–11974			
	RXN–8023			
	RXN–8024			
	RXN–8022			
	RXN–12412			
LycE (lycopene ε–cyclase)	RXN–8040		7 sequences (ORTHOLOGY x2)	18 sequences (ORTHOLOGY x3)
LycB (lycopene β–cyclase)	RXN–8038			
LycE (lycopene ε–cyclase)	RXN1F–147			
	RXN–8028			
LycB (lycopene β–cyclase)	RXN1F–148			
Carotene epsilon monooxygenase	RXN–5961		5 sequences (ORTHOLOGY)	7 sequences (ORTHOLOGY x2)
	RXN–5962		6 sequences (ORTHOLOGY)	8 sequences (ORTHOLOGY x2)
	RXN–12226		—	g13263.t1 (ANNOTATION)
Reactions added manually				
DDE (diadinoxanthin de–epoxidase)	RXN–19200	[12,59,61]	SJ20456 (VDE–like ~ DDE)	g16187 (VDE–like ~ DDE)
DEP (diatoxanthin–epoxidase)	RXN–19202		—	—
1.3.99. –	RXN–19203	[5,18,60]	—	—
1.14.99.M8	RXN–19197		—	—
2.3.1. –	RXN–19196		—	—
—	RXN–19184	[60]	—	—
—	RXN1F–155		—	—
—	ConversionViolaxanthinToDiadinoxanthin	[18]	—	—
—	ConversionDiadinoxanthinToFucoxanthin	[18]	—	—
