# Supplementary material for: Genome–Scale Metabolic Networks Shed Light on the Carotenoid Biosynthesis Pathway in the Brown Algae Saccharina japonica and Cladosiphon okamuranus
Source: Antioxidants (Basel). 2019 Nov 16;8(11):564. doi: 10.3390/antiox8110564 (PMC6912245; doi:10.3390/antiox8110564)
Supplement: Supplementary file 1 [file antioxidants-08-00564-s001.pdf]

Genome-Scale Metabolic Networks Shed Light on the Carotenoid Biosynthesis Pathway in the Brown Algae *Saccharina japonica* and *Cladosiphon okamuranus*

Delphine Nègre[1,2,3], Méziane Aite[4], Arnaud Belcour[4], Clémence Frioux[4,5], Loraine Brillet–Guéguen[1,2], Xi Liu[2], Philippe Bordron[2], Olivier Godfroy[1], Agnieszka P. Lipinska[1], Catherine Leblanc[1], Anne Siegel[4], Simon M. Dittami[1], Erwan Corre[2] and Gabriel V. Markov[1*]

[1] Sorbonne Université, CNRS, Integrative Biology of Marine Models (LBI2M), Station Biologique de Roscoff (SBR), 29680 Roscoff, France; delphine.negre@sb-roscoff.fr (D.N.); loraine.gueguen@sb-roscoff.fr (L.B.-G.); olivier.godfroy@sb-roscoff.fr (O.G.); alipinska@sb-roscoff.fr (A.P.L.); catherine.leblanc@sb-roscoff.fr (C.L.); simon.dittami@sb-roscoff.fr (S.M.D.); gabriel.markov@sb-roscoff.fr (G.V.M)

[2] Sorbonne Université, CNRS, Plateforme ABiMS (FR2424), Station Biologique de Roscoff, 29680 Roscoff, France; xi.liu@sb-roscoff.fr (X.L.); philippe.bordron@univ-nantes.fr (P.B.); erwan.corre@sb-roscoff.fr (E.C.)

[3] Groupe Mer, Molécules, Santé-EA 2160, UFR des Sciences Pharmaceutiques et Biologiques, Université de Nantes, 9, Rue Bias, 44035 Nantes, France

[4] Université de Rennes 1, Institute for Research in IT and Random Systems (IRISA), Equipe Dyliss, 35052 Rennes, France; meziane.aite@inria.fr (M.A.); arnaud.belcour@irisa.fr (A.B.); clemence.frioux@quadram.ac.uk (C.F.); anne.siegel@irisa.fr (A.S.)

[5] Quadram Institute, Colney Lane, Norwich, NR4 7UQ, United Kingdom

[*] Correspondence: gabriel.markov@sb-roscoff.fr

Contents

Table S1: Data from the annotation of algal protein-coding genes. ... 2

Figure S1: Source of the *S. japonica* (a) and *C. okamuranus* (b) annotations ... 3

Table S2: Targets for *S. japonica* from metabolic data in the literature. ... 6

Table S3: Targets for *C. okamuranus* from metabolic data in the literature. ... 9

Table S4: List of cofactors (seed) added to the *S. japonica* and *C. okamuranus* GSMNs. ... 10

Table S5: List of biomass reactions added to the *S. japonica* and *C. okamuranus* GSMNs. ... 11

Table S6: Accession number of enzymes and protein sequences used. ... 12

Figure S2: Pathways of methylerythritol phosphate (MEP) within the *S. japonica* and *C. okamuranus* GSMNs. ... 13

Table S7: List of reactions and genes associated with MEP pathways in the GSMNs of three brown algae: *S. japonica*, *C. okamuranus* and *E. siliculosus* ... 14

Figure S3: Pathways of mevalonate (MVA) within the *S. japonica* and *C. okamuranus* GSMNs. ... 15

Table S8: List of reactions and genes associated with MVA pathways in the GSMNs of three brown algae: *S. japonica*, *C. okamuranus* and *E. siliculosus* ... 16

Figure S4: Maximum likelihood tree of the lycopene cyclase family. ... 17

Figure S5: Maximum likelihood tree of the NCED family. ... 20

Figure S6: Maximum likelihood tree of the xanthine oxidase family. ... 21

Table S9: Type of SDR found in the genomes of *S. japonica* and *C. okamuranus*. ... 23

Figure S7: Maximum likelihood tree of the Mocos family. ... 24

Figure S8: Maximum likelihood tree of the VDE. ... 26

Figure S9: Amino acid length of unannotated coding sequences of *S. japonica* (a) and *C. okamuranus* (b). 28

Figure S10: Enrichment of the annotation in GO terms and KEGG identifiers: data fusion. ... 29

	S. japonica	*C. okamuranus*
**Data after cleaning**		
• Number of scaffolds	12,993	540
• Average sequence length (residue counts)	39,875	240,150
• Minimum sequence length (nucleotides)	500	2,074
• Maximum sequence length (nucleotides)	1,469,055	2,792,633
**Potential coding sequences (Transdecoder)**	17,861	15,140
• Average sequence length (residue counts)	511.38	659.92
• Minimum sequence length (amino acids)	49	49
• Maximum sequence length (amino acids)	13,870	1, 363
**Protein domain research (Hmmscan)**		
• Total number of protein domains detected	23,617	18,480
• Number of different protein domains detected	3,498	3,520
• Number of sequences annotated by at least one protein domain	9,826	9,390
**Nature of proteins**		
• Sequences with transmembrane helices (TmHMM)	3,229	2,963
• Sequences with signal peptide (SignalP)	2,059	1,114
**Gene Ontology Term (GOT)**		
• Number of sequences annotated by at least one GOT (Trinotate)	9,532	9,479
• Number of sequences annotated by at least one GOT (Blast2GO)	8,269	-
• Number of sequences annotated by at least one GOT (fusion)	9,663	-
• Total number of GOT detected	589,808	584,589
• Number of different GOT detected	13,700	13,468
**Kyoto Encyclopedia of Genes and Genomes (KEGG)**		
• Number of sequences annotated by at least one KEGG (Trinotate)	5,317	5,723
• Number of sequences annotated by at least one KEGG (Kobas)	3,620	-
• Number of sequences annotated by at least one KEGG (fusion)	5,805	-
• Total number of KEGG detected	5,940	5,723
• Number of different KEGG detected	3,430	3,245
**Enzyme Commission numbers (EC)**		
• Number of sequences annotated at least by one EC	2,575	2,505
• Total number of EC detected	2,958	2,769
• Number of different EC detected	1,093	995

Table S1: Data from the annotation of algal protein-coding genes.

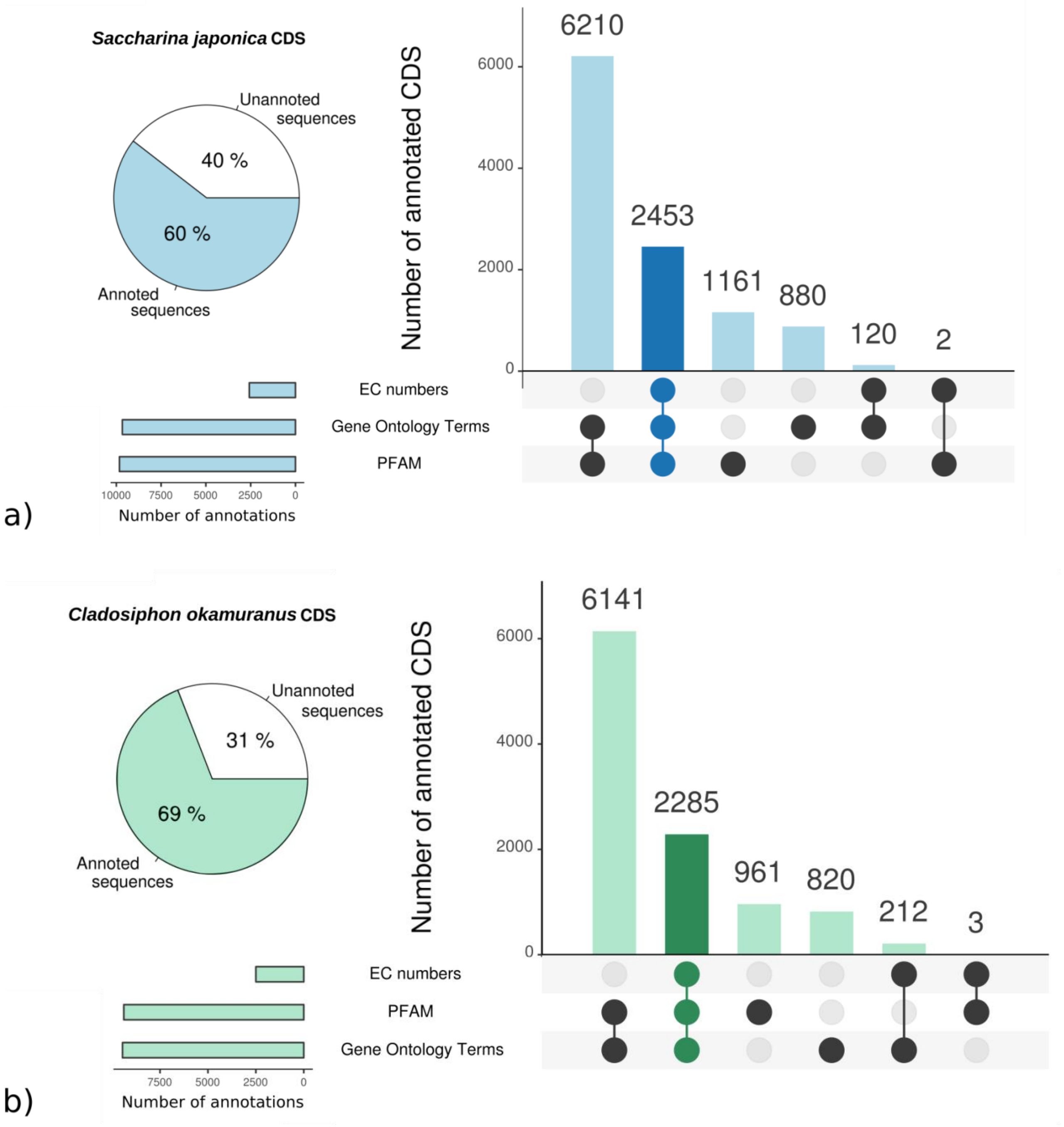

Figure S1: Source of the *S. japonica* (a) and *C. okamuranus* (b) annotations. Upset plots represent the distribution of the three types of annotation data: EC numbers, gene ontology terms (GOT) and protein domains (PFAM) for all predicted coding sequences.

In order to provide a first source of 'annotation-based' metabolic reactions, EC numbers, Gene ontology terms (GOT), and protein domains (PFAM) were sought among the predicted coding sequences of *S. japonica* and *C. okamuranus*. 60% (10,826 out of 17,898) and 69% (10,422 out of 15,077) of them were annotated with at least one of these three types of annotations and 22% of them are annotated by all three data types. These annotations allowed the generation of GSMNs composed respectively of 4,316 and 4,003 genes, 1,237 and 1,205 pathways, 2,560 and 2,448 enzymatic reactions and 2,920 and 2,872 metabolites for *S. japonica* and *C. okamuranus*.

Usual name	Chemical Formula	MetaCyC ID	Reference	Reachable after gap-filling
Alginate	$(C_6H_8O_6)_n$	ALGINATE	[1]	+
Eicosanoic acid / archidate	$C_{20}H_{39}O_2$	ARACHIDIC_ACID	[2]	+
Arachidonic acid / arachidonate	$C_{20}H_{31}O_2$	ARACHIDONIC_ACID	[2]	+
Arginine	$C_6H_{15}N_4O_2$	ARG	[3]	+
Asparagine	$C_4H_8N_2O_3$	ASN	[3]	+
Cholesterol	$C_{27}H_{46}O$	CHOLESTEROL	[4]	+
β-L-Fucose / β-L-fucopyranose	$C_6H_{12}O_5$	CPD0-1107	[1]	
Acid docosahexaenoic / docosahexaenoate	$C_{22}H_{31}O_2$	CPD-10244	[3]	+
Erucic acid / erucate	$C_{22}H_{41}O_2$	CPD-14292	[3]	
Heptadecenoic acid	$C_{17}H_{31}O_2$	CPD-15680	[2]	
Fructose-6-P	$C_6H_{11}O_9P$	CPD-15709	[1]	+
Eicosatrienoic acid /sciadonate	$C_{20}H_{33}O_2$	CPD-17350	[2]	
GDP-a-D-mannuronate	$C_{16}H_{20}N_5O_{17}P_2$	CPD-1828	[5]	+
Caprylic acid / octanoate	$C_8H_{15}O_2$	CPD-195	[3]	+
Tridecanoic acid / tridecanoate	$C_{13}H_{25}O_2$	CPD-19659	[3]	
Capric acid / decanoate	$C_{10}H_{19}O_2$	CPD-3617	[3]	+
Isofucosterol	$C_{29}H_{48}O$	CPD-4127	[4]	+
Isopentenyl adenosine	$C_{15}H_{21}N_5O_4$	CPD-4207	[6]	+
β-L-fucose 1-phosphate	$C_6H_{11}O_8P$	CPD-488	[1]	
Abscissic acid	$C_{15}H_{19}O_4$	CPD-693	[7]	
24-Methylenecholesterol	$C_{28}H_{46}O$	CPD-706	[8]	+
Heptadecanoic acid	$C_{17}H_{33}O_2$	CPD-7830	[2]	+
Tricosanoic acid / tricosanoate	$C_{23}H_{45}O_2$	CPD-7834	[3]	
Myristic acid / myristate	$C_{14}H_{27}O_2$	CPD-7836	[2]	+
γ-linolenic acid	$C_{18}H_{29}O_2$	CPD-8117	[2]	
Di-homo-γ-linolenic acid / eicosatrienoic acid	$C_{20}H_{33}O_2$	CPD-8120	[3]	+

Pentadecanoic acid / pentadecanoate	$C_{15}H_{29}O_2$	CPD-8462	[2]	+
Palmitoleic acid / palmitoleate	$C_{16}H_{29}O_2$	CPD-9245	[3]	+
Eicosadienoic acid	$C_{20}H_{35}O_2$	CPD-9842	[2]	
Cysteine	$C_3H_7NO_2S$	CYS	[3]	+
Desmosterol	$C_{27}H_{44}O$	DESMOSTEROL-CPD	[4]	+
Behenic acid / behenate	$C_{22}H_{43}O_2$	DOCOSANOATE	[3]	
Lauric acid / laurate	$C_{12}H_{23}O_2$	DODECANOATE	[3]	+
GDP-α-D-mannose	$C_{16}H_{23}N_5O_{16}P_2$	GDP-MANNOSE	[1]	+
Glutaminic acid / L-glutamate	$C_5H_8NO_4$	GLT	[3]	+
Glycine	$C_2H_5NO_2$	GLY	[3]	+
Hexanoic acid/ hexanoate	$C_6H_{11}O_2$	HEXANOATE	[2]	+
Histidine	$C_6H_9N_3O_2$	HIS	[3]	+
Isoleucine	$C_6H_{13}NO_2$	ILE	[3]	+
Alanine	$C_3H_7NO_2$	L-ALPHA-ALANINE	[3]	+
Aspartic acid	$C_4H_6NO_4$	L-ASPARTATE	[3]	+
Leucine	$C_6H_{13}NO_2$	LEU	[3]	+
Linoleic acid / linoleate	$C_{18}H_{31}O_2$	LINOLEIC_ACID	[2]	+
Lysine	$C_6H_{15}N_2O_2$	LYS	[3]	+
Mannitol	$C_6H_{14}O_6$	MANNITOL	[1]	+
Mannitol-1-P	$C_6H_{13}O_9P$	MANNITOL-1P	[9]	+
Mannose-1-P	$C_6H_{11}O_9P$	MANNOSE-1P	[1]	+
Mannose-6-P	$C_6H_{11}O_9P$	MANNOSE-6P	[1]	+
Methionine	$C_5H_{11}NO_2S$	MET	[3]	+
Oleic acid / oleate	$C_{18}H_{33}O_2$	OLEATE-CPD	[2]	+
Palmitic acid	$C_{16}H_{31}O_2$	PALMITATE	[2]	+
Phenylalanine	$C_9H_{11}NO_2$	PHE	[3]	+
Mannuronan	$(C_6H_9O_7)_n$	Poly-beta-D-Mannuronate	[1]	+

Proline	$C_5H_9NO_2$	PRO	[3]	+
Serine	$C_3H_7NO_3$	SER	[3]	+
Stearic acid / Stearate	$C_{18}H_{35}O_2$	STEARIC_ACID	[2]	+
Tetracosanoic acid / lignocerate	$C_{24}H_{47}O_2$	TETRACOSANOATE	[2]	+
Threonine	$C_4H_9NO_3$	THR	[3]	+
Tryptophan	$C_{11}H_{12}N_2O_2$	TRP	[3]	+
Tyrosine	$C_9H_{11}NO_3$	TYR	[3]	+
Valine	$C_5H_{11}NO_2$	VAL	[3]	+
**Metabolites present but not targeted by gap-filling due to their encoding (type: compound class)**				
Xylose	$C_5H_{10}O_5$	D-L-Xylose	[10]	
Eicosadienoic acid / icosadienoate	$C_{20}H_{35}O_2$	Eicosadienoates	[3]	
Eicosatrienoic acid	$C_{20}H_{34}O_2$	Eicosatrienoates	[2]	
Fructose	$C_6H_{12}O_6$	FRU	[9]	
Fucan	$(C_6H_{10}O_4)_n$	Fucan	[1]	
Fucoidan	$C_7H_{14}O_7S$	Fucoidans	[1]	
Fucose	$C_6H_{12}O_5$	Fucoses	[10]	
Galactose	$C_6H_{12}O_6$	Galactose	[10]	
Glucose	$C_6H_{12}O_6$	Glucose	[10]	
L-guluronate	$C_6H_9O_7$	L-Guluronate	[5]	
Rhamnose	$C_6H_{12}O_5$	Rhamnose	[10]	

Table S2: Targets for *S. japonica* from metabolic data in the literature.

References

1. Chi, S.; Liu, T.; Wang, X.; Wang, R.; Wang, S.; Wang, G.; Shan, G.; Liu, C. Functional genomics analysis reveals the biosynthesis pathways of important cellular components (alginate and fucoidan) of Saccharina. *Curr Genet* **2017**, 1–15.
2. Park, J.-N.; Ali-Nehari, A.; Woo, H.-C.; Chun, B.-S. Thermal stabilities of polyphenols and fatty acids in *Laminaria japonica* hydrolysates produced using subcritical water. *Korean J. Chem. Eng.* **2012**, *29*, 1604–1609.
3. Getachew, P.; Kang, J.-Y.; Choi, J.-S.; Hong, Y.-K. Does bryozoan colonization alter the biochemical composition of *Saccharina japonica* affecting food safety and quality? *Botanica Marina* **2015**, *58*, 267–274.
4. Patterson, G.W. Sterols of *Laminaria*. *Comparative biochemistry and physiology* **1968**, *24*, 501–505.
5. Zhang, P.; Shao, Z.; Jin, W.; Duan, D. Comparative characterization of two GDP-mannose dehydrogenase genes from *Saccharina japonica* (*Laminariales, Phaeophyceae*). *BMC Plant Biology* **2016**, *16*.
6. Duan, D.; Liu, X.; Pan, F.; Liu, H.; Chen, N.; Fei, X. Extraction and Identification of Cytokinin from *Laminaria japonica Aresch*. *Botanica Marina* **1995**, *38*, 409–412.
7. Schaffelke, B. Abscisic Acid in Sporophytes of Three *Laminaria* Species (*Phaeophyta*). *Journal of Plant Physiology* **1995**, *146*, 453–458.
8. Honya, M.; Kinoshita, T.; Ishikawa, M.; Mori, H.; Nisizawa, K. Seasonal variation in the lipid content of cultured *Laminaria japonica*: fatty acids, sterols, β-carotene and tocopherol. *Journal of Applied Phycology* **1994**, *6*, 25–29.
9. Hwang, J.-H.; Kim, N.-G.; Woo, H.-C.; Rha, S.-J.; Kim, S.-J.; Shin, T.-S. Variation in the chemical composition of *Saccharina japonica* with harvest area and culture period. *Journal of Aquaculture Research & Development* **2014**, *5*, 1.
10. Groisillier, A.; Shao, Z.; Michel, G.; Goulitquer, S.; Bonin, P.; Krahulec, S.; Nidetzky, B.; Duan, D.; Boyen, C.; Tonon, T. Mannitol metabolism in brown algae involves a new phosphatase family. *J Exp Bot* **2014**, *65*, 559–570.

Usual name	Chemical Formula	MetaCyC ID	Reference	Reachable after gap-filling
(5Z)-tetradecenoyl-CoA	14:1(n-9) fatty acid	CPD-15436	[1]	+
Acetic acid	$C_2H_3O_2$	ACET	[2]	+
Alginate	$(C_6H_8O_6)_n$	ALGINATE	[2]	+
Alpha Linolenic acid	18:3(n-3) fatty acid	LINOLENIC_ACID	[1]	
Arachidonic acid	20:4(n-6) fatty acid	ARACHIDONIC_ACID	[1]	+
D-glucuronic acid	$C_6H_7O_7$	GLUCURONATE	[2]	
Docosanoic acid	22:0 fatty acid	DOCOSANOATE	[1]	
Dodecanoic acid	12:0 fatty acid	DODECANOATE	[1]	+
Eicosanoic acid	20:0 fatty acid	ARACHIDIC_ACID	[1]	+
Eicosapentaenoic acid (EPA)	20:5(n-3) fatty acid	5Z8Z11Z14Z17Z-EICOSAPENTAENOATE	[1,3]	+
Glycerate	$C_3H_5O_4$	GLYCERATE		+
Heptadecanoic acid	17:0 fatty acid	CPD-7830	[1]	+
Histidine	$C_6H_9N_3O_2$	HIS		+
Leucine	$C_6H_{13}NO_2$	LEU		+
Linoleic acid	18:2(n-6) fatty acid	LINOLEIC_ACID	[1]	+
Mannitol	$C_6H_{14}O_6$	MANNITOL		+
Myristic acid	14:0 fatty acid	CPD-7836	[1]	+
Octadecatetraenoic acid	18:4(n-3) fatty acid	CPD-12653	[1,4]	
Oleic acid	18:1(n-9) fatty acid	OLEATE-CPD	[1]	+
Palmitic acid	16:0 fatty acid	PALMITATE	[1]	+
Palmitoleic acid	16:1(n-7) fatty acid	CPD-9245	[1]	+
Pentadecanoic acid	15:0 fatty acid	CPD-8462	[1]	+
Phytol	acyclic diterpene alcohol	PHYTOL	[3]	+
Sapienate	16:1(n-10) fatty acid	CPD-14393	[1]	
Stearic acid	18:0 fatty acid	STEARIC_ACID	[1]	+

Tetracosanoic acid	24:0 fatty acid	TETRACOSANOATE	[1]	+
Tridecanoate	13:0 fatty acid	CPD-19659	[1]	
Valine	$C_5H_{11}NO_2$	VAL		+
γ-linolenic acid	18:3(n-6) fatty acid	CPD-8117	[1]	

**Metabolites present but not targeted by gap-filling due to their encoding (type: compound class)**				
D-xylose	$C_5H_{10}NO_5$	D-Xylose	[2]	
Fucoidan	$C_7H_{14}O_7S$	Fucoidans	[5]	
L-fucose	$C_6H_{12}O_5$	L-fucoses	[2]	
Uronic acid	$C_6H_{10}O_7$	Uronates	[2]	

Table S3: Targets for *C. okamuranus* from metabolic data in the literature.

References

1. Saito, H.; Xue, C.; Yamashiro, R.; Moromizato, S.; Itabashi, Y. High Polyunsaturated Fatty Acid Levels in Two Subtropical Macroalgae, *Cladosiphon Okamuranus* and *Caulerpa Lentillifera*. *Journal of Phycology* **2010**, *46*, 665–673.
2. Tako, M.; Yoza, E.; Tohma, S. Chemical Characterization of Acetyl Fucoidan and Alginate from Commercially Cultured *Cladosiphon okamuranus*. *Botanica Marina* **2000**, *43*.
3. Cheng, K.-C.; Kuo, P.-C.; Hung, H.-Y.; Yu, K.-H.; Hwang, T.-L.; Shieh, P.-C.; Chang, J.-S.; Wu, T.-S. Four new compounds from edible algae *Cladosiphon okamuranus* and *Chlorella sorokiniana* and their bioactivities. *Phytochemistry Letters* **2016**, *18*, 113–116.
4. Lim, S.J.; Wan Aida, W.M.; Schiehser, S.; Rosenau, T.; Böhmdorfer, S. Structural elucidation of fucoidan from *Cladosiphon okamuranus* (Okinawa mozuku). *Food Chemistry* **2019**, *272*, 222–226.
5. Kakisawa, H.; Asari, F.; Kusumi, T.; Toma, T.; Sakurai, T.; Oohusa, T.; Hara, Y.; Chiharai, M. An allelopathic fatty acid from the brown alga *Cladosiphon okamuranus*. *Phytochemistry* **1988**, *27*, 731–735.

Usual name	MetaCyC ID	Usual name	MetaCyC ID
Acyl-carrier protein	ACP	Guanosine 5'-triphosphate	GTP
Adenosylcobalamin	ADENOSYLCOBALAMIN	Hydrogen peroxide	HYDROGEN-PEROXIDE
Adenosine-5'-diphosphate	ADP	Potassium ion	K+
Ammonia	AMMONIA	Hν	Light
Adenosine 5'-monophosphate	AMP	Magnesium ion	MG+2
Adenosine-5'-triphosphate	ATP	Manganese ion	MN+2
Oxidized electron acceptor	Acceptor	Sodium ion	NA+
Vitamin H / coenzyme R	BIOTIN	Nicotinamide adenine dinucleotide	NAD
Calcium	CA+2	NAD(P)	NAD-P-OR-NOP
Carbon dioxide	CARBON-DIOXIDE	Nicotinamide adenine dinucleotide reduced	NADH
Chloride	CL-	NAD(P)H	NADH-P-OR-NOP
Cobalt	CO+2	Nicotinamide adenine dinucleotide phosphate	NADP
Cobalamin	COB-I-ALAMIN	Reduced nicotinamide adenine dinucleotide phosphate	NADPH
Molybdate	CPD-3	Nitrate	NITRATE
Vitamine B12 / cyanocobalamin	CPD-315	Oxygen	OXYGEN-MOLECULE
Cuprous ion	CU+	Diphosphate	PPI
Cupric ion	CU+2	Hydrogen ion	PROTON
Reduced electron acceptor	Donor-H2	Phosphate	Pi
Ethylenediaminetetraacetic acid	EDTA	Sulfuric acid	SULFATE
Ferrous iron	FE+2	Vitamine B1 / thiamine	THIAMINE
Ferric iron	FE+3	Uridine-5'-diphosphate	UDP
Oxidized cytochrome b_5	FERRICYTOCHROME-B5	H2O	WATER
Reduced cytochrome b_5	FERROCYTOCHROME-B5	Zinc ion	ZN+2
Guanosine-5'-diphosphate	GDP		

Table S4: List of cofactors (seed) added to the *S. japonica* and *C. okamuranus* GSMNs.

reaction_id biomass_rxn
comment forFBA
reversible false
linked_gene None
#reactant/product #stoichio:compound_id
reactant 0.01:4-AMINO-BUTYRATE:c
reactant 0.1:ARG:c
reactant 0.3:MET:c
reactant 0.75:ASN:c
reactant 19.3055:CIT:c
reactant 0.3:CYS:c
reactant 2.7615:GLC:c
reactant 10.7:GLN:c
reactant 18.85:GLT:c
reactant 3.8:GLY:c
reactant 0.123:GLYCERATE:c
reactant 2.375:GLYCEROL:c
reactant 0.029:GLYCOLLATE:c
reactant 0.1:HIS:c
reactant 0.2:ILE:c
reactant 26.6:L-ALPHA-ALANINE:c
reactant 12.5:L-ASPARTATE:c
reactant 0.1:L-ORNITHINE:c
reactant 0.3:LEU:c
reactant 0.35:LYS:c
reactant 331.4:MANNITOL:c
reactant 0.35:PHE:c
reactant 1.35:PRO:c
reactant 3.35:SER:c
reactant 0.878:SUC:c
reactant 0.65:THR:c
reactant 9.37:THREO-DS-ISO-CITRATE:c
reactant 0.115:TRP:c
reactant 0.125:TYR:c
reactant 0.85:VAL:c
product 1:BIOMASS:c

reaction_id Transport_BIOMASS
comment transport biomass to external compartment
reversible false
linked_gene
#reactant/product
reactant 1:BIOMASS:c
product 1:BIOMASS:e

reaction_id Exchange_BIOMASS
comment Exchange biomass to boundary
reversible false
linked_gene
#reactant/product
reactant 1:BIOMASS:e
product 1:BIOMASS:C-BOUNDARY

Table S5: List of biomass reactions added to the *S. japonica* and *C. okamuranus* GSMNs.

**Carotenoid biosynthesis pathway and the first cycle of xanthophylls**		
ORCAE: Online Resource for Community Annotation of Eukaryotes		
Enzyme	Organism	Accesion numbers
Phytoene synthase (**PSY**)	*Ectocarpus siliculosus*	Ec-04_002810.1
Phytoene desaturase (**PDS**)	*Ectocarpus siliculosus*	Ec-18_001850.1
ζ-carotene desaturase (**ZDS**)	*Ectocarpus siliculosus*	Ec-02_004660.1
Prolycopene isomerase (**crtISO**)	*Ectocarpus siliculosus*	Ec-20_003200.1
Cytochrome P450 (**Cyp97E3**)	*Ectocarpus siliculosus*	Ec-14_006690.1
Cytochrome P450 (**Cyp97F4**)	*Ectocarpus siliculosus*	Ec-14_006700.1
Zeaxanthin epoxidase (**ZEP**)	*Ectocarpus siliculosus*	Ec-22_000820.1
Uniprot database		
Enzyme	Organism	Accesion numbers
ζ-carotene isomerase (**Z-iso**)	*Arabidopsis thaliana*	Q9SAC0
Lycopene β-cyclase (**LYCB**)	*Arabidopsis thaliana*	Q38933
Lycopene ε-cyclase (**LYCE**)	*Arabidopsis thaliana*	Q38932
Violaxanthin de-epoxidase (**VDE**)	*Arabidopsis thaliana*	Q39249

**Biosynthesis of acid abscisic**		
Uniprot database		
Enzyme	Organism	Accesion numbers
Neoxanthin synthase, chloroplastic (**Nsy**)	*Solanum Liycopersicum*	K4C9E2
9-cis-epoxycarotenoid dioxygenase (**NCED3**)	*Arabidopsis thaliana*	Q9LRR7
9-cis-epoxycarotenoid dioxygenase (**NCED1**)	*Zea Maize*	O24592
9-cis-epoxycarotenoid dioxygenase (**NCED1**)	*Solanum lycopersicum*	O24023
Xanthoxin dehydrogenase (**ABA2**)	*Arabidopsis thaliana*	Q9C826
Abscisic-aldehyde oxidase (**AAO3**)	*Arabidopsis thaliana*	Q7G9P4
Molybdenum cofactor sulfurase (**ABA3**)	*Arabidopsis thaliana*	Q9C5X8
Abscisate β -glucosyltransferase (**AOG**)	*Vigna angularis*	Q8W3P8
B-D-glucopyranosyl abscisate β -glucosidase (**BG1**)	*Arabidopsis thaliana*	Q9SE50

**Proteomes**		
Organism	Accesion numbers (ID)	Database
Arabidopsis thaliana	ID 4	NCBI
Chlamydomonas reinhardtii	ID 147	NCBI
Chondrus crispus	ID 12106	NCBI
Cladosiphon okamuranus	-	Publication (Nishitsuji et al, 2016)
Cyanidioschyzon merolae	ID 79	NCBI
Ectocarpus siliculosus	*Ectocarpus siliculosus* V2	ORCAE
Galdieria sulphuraria	ID 405	NCBI
Nannochloropsis gaditana	ID 11691	NCBI
Nannochloropsis salina	-	Publication (Loira et al, 2017)
Phaeodactylum tricornutum	ID 418	NCBI
Porphyra umbilicalis	ID 12861	NCBI
Saccharina japonica	-	Publication (Ye et al, 2015)
Ulva mutabilis	*Ulva mutabilis*	ORCAE
Volvox carteri	ID 413	NCBI

Table S6: Accession number of enzymes and protein sequences used.

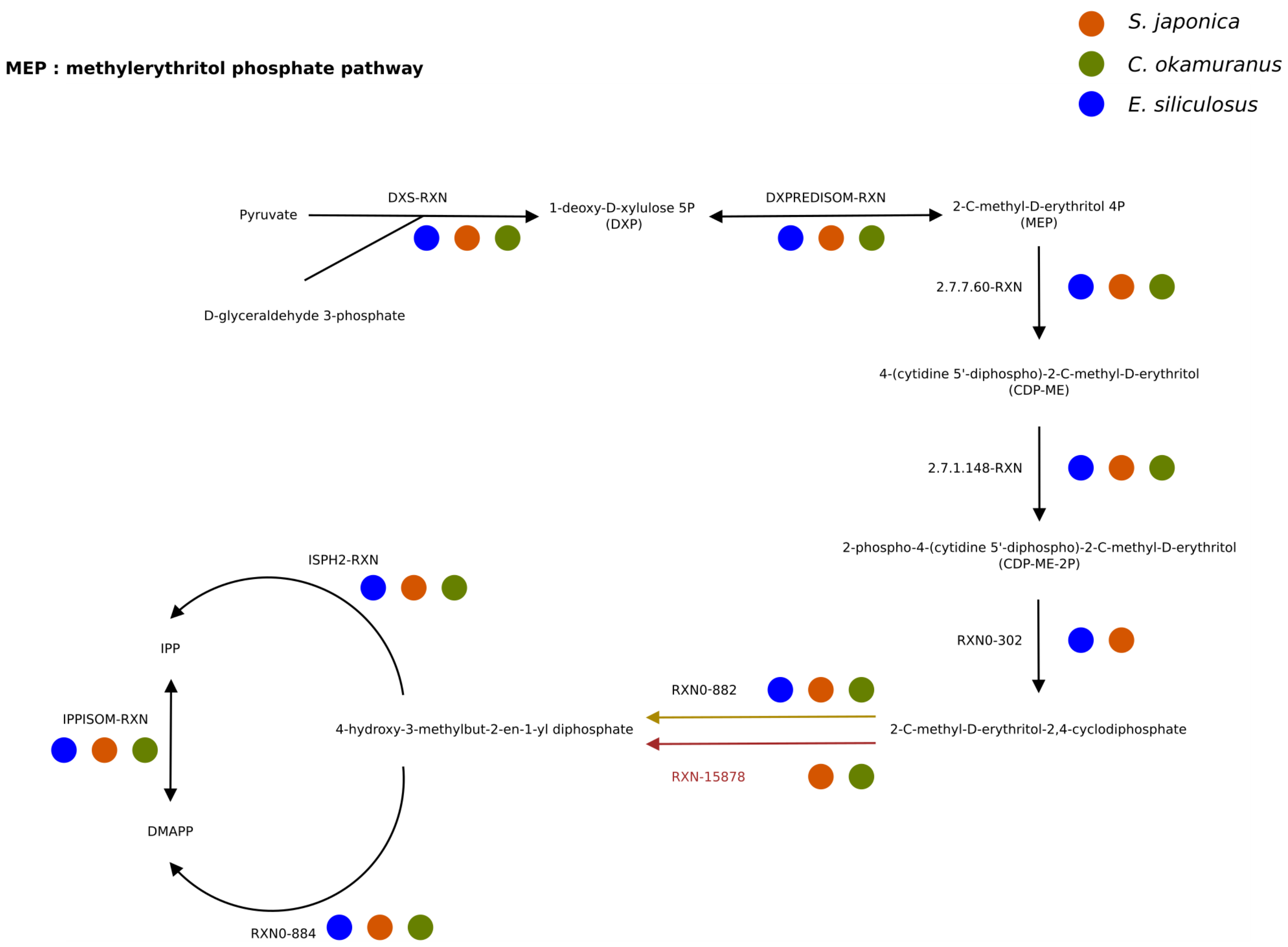

Figure S2: Pathways of methylerythritol phosphate (MEP) within the *S. japonica* and *C. okamuranus* GSMNs. According to [1] and the MetaCyc database [2] pathways NONMEVIPP-PWY (methylerythritol phosphate pathway I) and PWY-7560 (methylerythritol phosphate pathway II). These two pathways are composed of nine reactions (all present in *S. japonica*, RXN0-302 absent in *C. okamuranus*) including eight in common. RXN0-882 (yellow) belongs to PWY-7560 and RXN-15878 (red) belongs to NONMEVIPP-PWY.

MEP: methylerythritol phosphate pathway				
**Steps/enzymes**	**Reactions**	***S. japonica***	***C. okamuranus***	***E. siliculosus***
1-deoxyxylulose-5-phosphate synthase	DXS-RXN	SJ15200; SJ12529	g7003; g14829	Ec-15_004230
1-deoxy-D-xylulose 5-phosphate reductoisomerase	DXPREDISOM-RXN	SJ11908	g11368; g11696	Ec-12_007940
4-diphosphocytidyl-2C-methyl-D-erythritol	2.7.7.60-RXN	SJ10622	g11729	Ec-12_008050
4-diphosphocytidyl-2-C-methylerythritol kinase	2.7.1.148-RXN	SJ02470	g16427	Ec-10_001370
2C-methyl-D-erythritol 2,4-cyclodiphosphate synthase	RXN0-302	SJ21110	-	Ec-25_000990
4-hydroxy-3-methylbut-2-en-1-yl diphosphate synthase	RXN0-882	SJ05642	g13905; g9095	Ec-03_003610
1-hydroxy-2-methyl-2-(E)-butenyl 4-diphosphate synthase	RXN-15878	SJ05642	g13905	-
Dimethylallyl-diphosphate:NAD(P)+ oxidoreductase	RXN0-884	SJ04795	g9095	Ec-06_002680
Isopentenyl-diphosphate:NAD(P)+ oxidoreductase	ISPH2-RXN	SJ04795	g9095	Ec-06_002680
Isopentenyl diphosphate isomerase	IPPISOM-RXN	SJ13532; SJ15203	g12669; g15492	Ec-11_001030; Ec-18_002690

Table S7: List of reactions and genes associated with the MEP pathways in the GSMNs of three brown algae: *S. japonica*, *C. okamuranus* and *E. siliculosus*. According to [1] and the MetaCyc database [2] pathways NONMEVIPP-PWY (methylerythritol phosphate pathway I) and PWY-7560 (methylerythritol phosphate pathway II). For colors, refer to Figure S2.

References

1. Zhao, L.; Chang, W.; Xiao, Y.; Liu, H.; Liu, P. Methylerythritol Phosphate Pathway of Isoprenoid Biosynthesis. *Annu. Rev. Biochem.* **2013**, *82*, 497–530.
2. Caspi, R.; Billington, R.; Fulcher, C.A.; Keseler, I.M.; Kothari, A.; Krummenacker, M.; Latendresse, M.; Midford, P.E.; Ong, Q.; Ong, W.K.; et al. The MetaCyc database of metabolic pathways and enzymes. *Nucleic Acids Res.* **2018**, *46*, D633–D639.

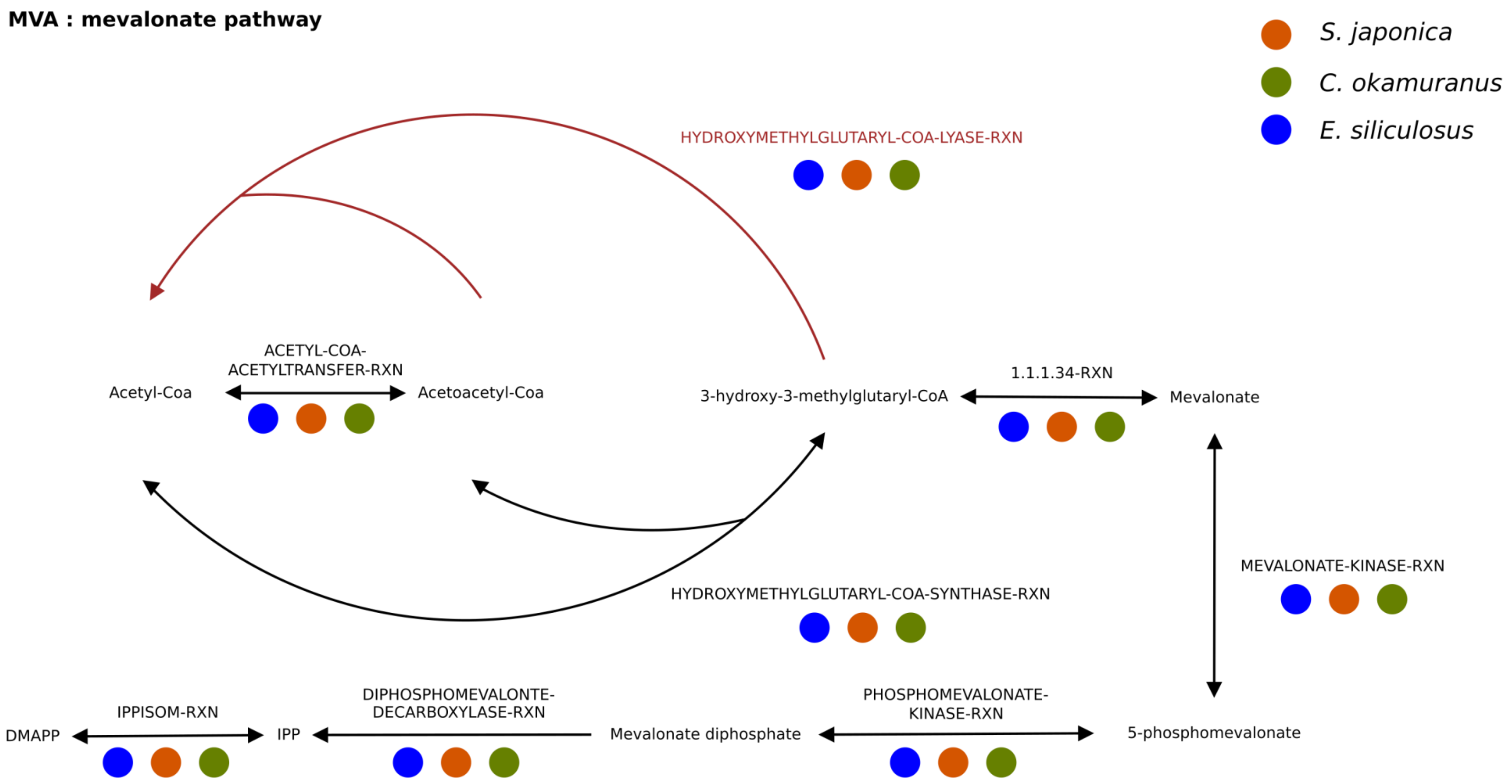

Figure S3: Pathways of mevalonate (MVA) within the *S. japonica* and *C. okamuranus* GSMNs. According to [1] and the MetaCyc database pathways PWY-922 (mevalonate pathway I) and PWY-5074 (mevalonate degradation). The synthetic pathways of mevalonate, composed of seven black reactions, is complete. In red, one of the two degradation reactions of mevalonate.

MVA: mevalonate pathway				
**Steps/enzymes**	**Reactions**	***S. japonica***	***C. okamuranus***	***E. siliculosus***
Acetyl-CoA C-acetyltransferase	ACETYL-COA-ACETYLTRANSFER-RXN	SJ21818; SJ09371; SJ14898	g13201; g7984; g14099	Ec-26_003940; Ec-24_000870; Ec-22_002850
Hydroxymethyl glutaryl-CoA synthase	HYDROXYMETHYLGLUTARYL-COA-SYNTHASE-RXN	SJ11593; SJ06617	g4145; g4023	Ec-19_002030; Ec-21_004360
Hydroxymethylglutaryl-CoA lyase	HYDROXYMETHYLGLUTARYL-COA-LYASE-RXN	SJ21564	g7646	Ec-26_006120
Hydroxymethylglutaryl-CoA reductase	1.1.1.34-RXN	SJ18136; SJ05150; SJ18779	g5043; g5042; g11410; g11409; g13444	Ec-03_000570; Ec-03_000580; Ec-08_002990; Ec-27_005710
Mevalonate kinase	MEVALONATE-KINASE-RXN	SJ11576; SJ09599	g15583	Ec-26_004370
Phosphomevalonate kinase	PHOSPHOMEVALONATE-KINASE-RXN	SJ04742	g9718	Ec-25_002910
Diphosphomevalonate decarboxylase	DIPHOSPHOMEVALONTE-DECARBOXYLASE-RXN	SJ05356	g12830	Ec-21_001440
Isopentenyl diphosphate isomerase	IPPISOM-RXN	SJ13532; SJ15203	g12669; g15492	Ec-11_001030; Ec-18_002690

Table S8: List of reactions and genes associated with MVA pathways in the GSMNs of three brown algae: *S. japonica*, *C. okamuranus* and *E. siliculosus*. According to [1] and the MetaCyc database pathways PWY-922 (mevalonate pathway I) and PWY-5074 (mevalonate degradation) [2]. For colors, refer to figure S3.

References

1. Zhao, L.; Chang, W.; Xiao, Y.; Liu, H.; Liu, P. Methylerythritol Phosphate Pathway of Isoprenoid Biosynthesis. *Annu. Rev. Biochem.* **2013**, *82*, 497–530.
2. Caspi, R.; Billington, R.; Fulcher, C.A.; Keseler, I.M.; Kothari, A.; Krummenacker, M.; Latendresse, M.; Midford, P.E.; Ong, Q.; Ong, W.K.; et al. The MetaCyc database of metabolic pathways and enzymes. *Nucleic Acids Res.* **2018**, *46*, D633–D639.

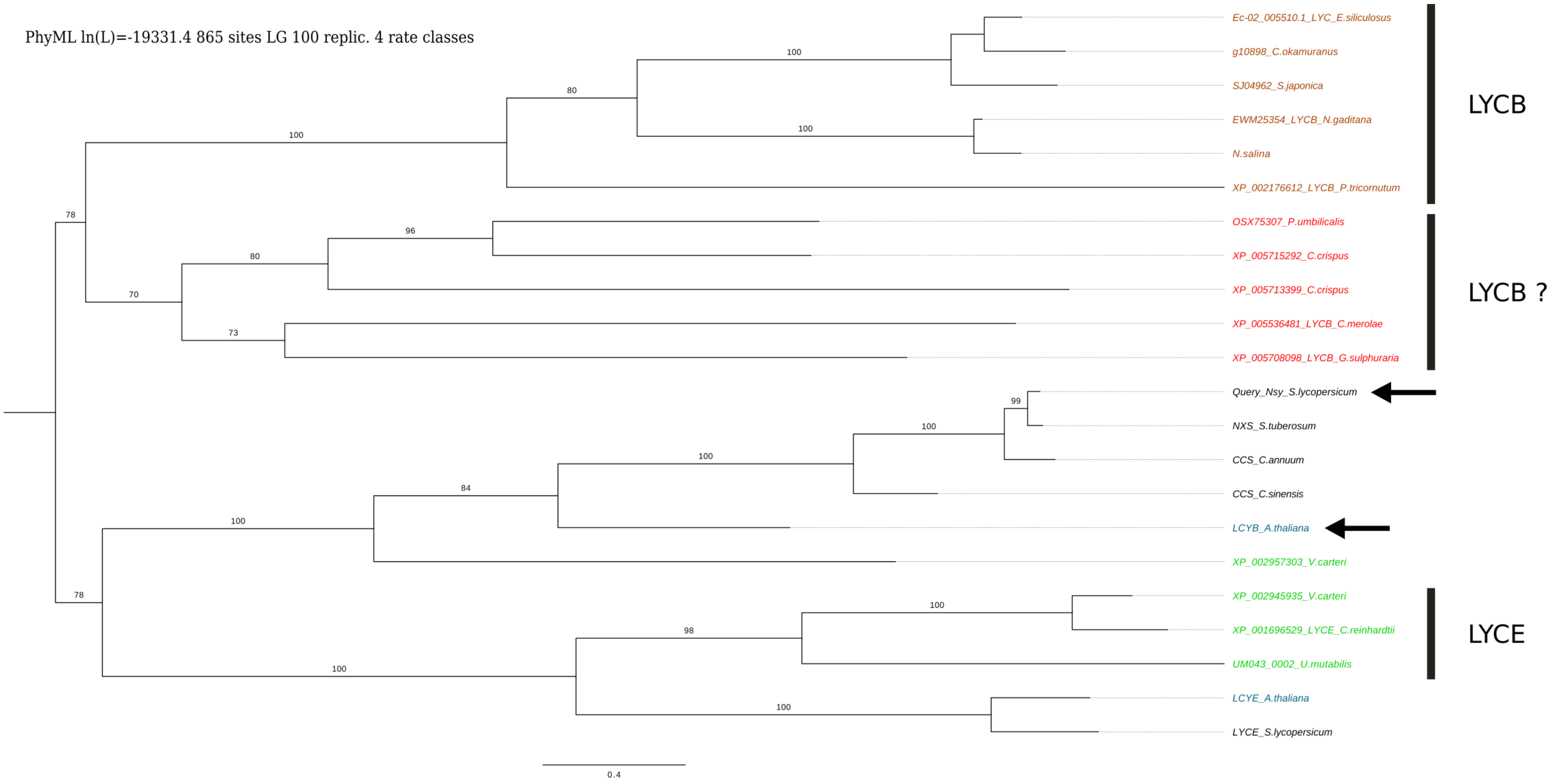

Figure S4: Maximum likelihood tree of the lycopene cyclase family. All the sequences provided with an accession number came from the NCBI database. The sequences presented on the various trees were selected based either on homology searches with the sequences identified QUERY (black arrow) or based on publications. When available, the functional annotation of the sequences were added to the sequence names. Stramenopile sequences (brown algae and diatoms) are shown in brown, red algae sequences in red, green algae sequences in green and *Arabidopsis thaliana* sequences in blue. Only bootstrap values greater than or equal to 70 are displayed. Tree were rooted by midpoint.

In terrestrial plants, neoxanthin is the last product of carotenoid synthesis and therefore the precursor of new oxygenated carotenoids. The enzyme responsible for this transformation, Nsy, belongs to the lycopene cyclase family characterized by the presence of the protein domain of the same name (PF05834). This carotenoid cyclase paralog has apparently been redesigned from the β-lycopene cyclase from terrestrial plants [1,2]. Homology search based on the Nsy sequence of *A. thaliana* on brown algae predicted protein sequences and the search for the protein domain in annotation files revealed a single candidate, identified as lycopene β-cyclase. The results obtained for red algae *C. merolae*, diatom *P. tricornutum* and green algae *C. reinhardtii* and *V. carteri* are similar to those obtained in [3].

References

1. Bouvier, F.; D'Harlingue, A.; Backhaus, R.A.; Kumagai, M.H.; Camara, B. Identification of neoxanthin synthase as a carotenoid cyclase paralog: Plastid neoxanthin synthase. *Eur. J. Biochem.* **2000**, *267*, 6346–6352.
2. Al-Babili, S.; Hugueney, P.; Schledz, M.; Welsch, R.; Frohnmeyer, H.; Laule, O.; Beyer, P. Identification of a novel gene coding for neoxanthin synthase from *Solanum tuberosum*. *FEBS Lett.* **2000**, *485*, 168–172.
3. Cui, H.; Wang, Y.; Qin, S. Molecular Evolution of Lycopene Cyclases Involved in the Formation of Carotenoids in Eukaryotic Algae. *Plant Mol. Biol. Report.* **2011**, *29*, 1013–1020.

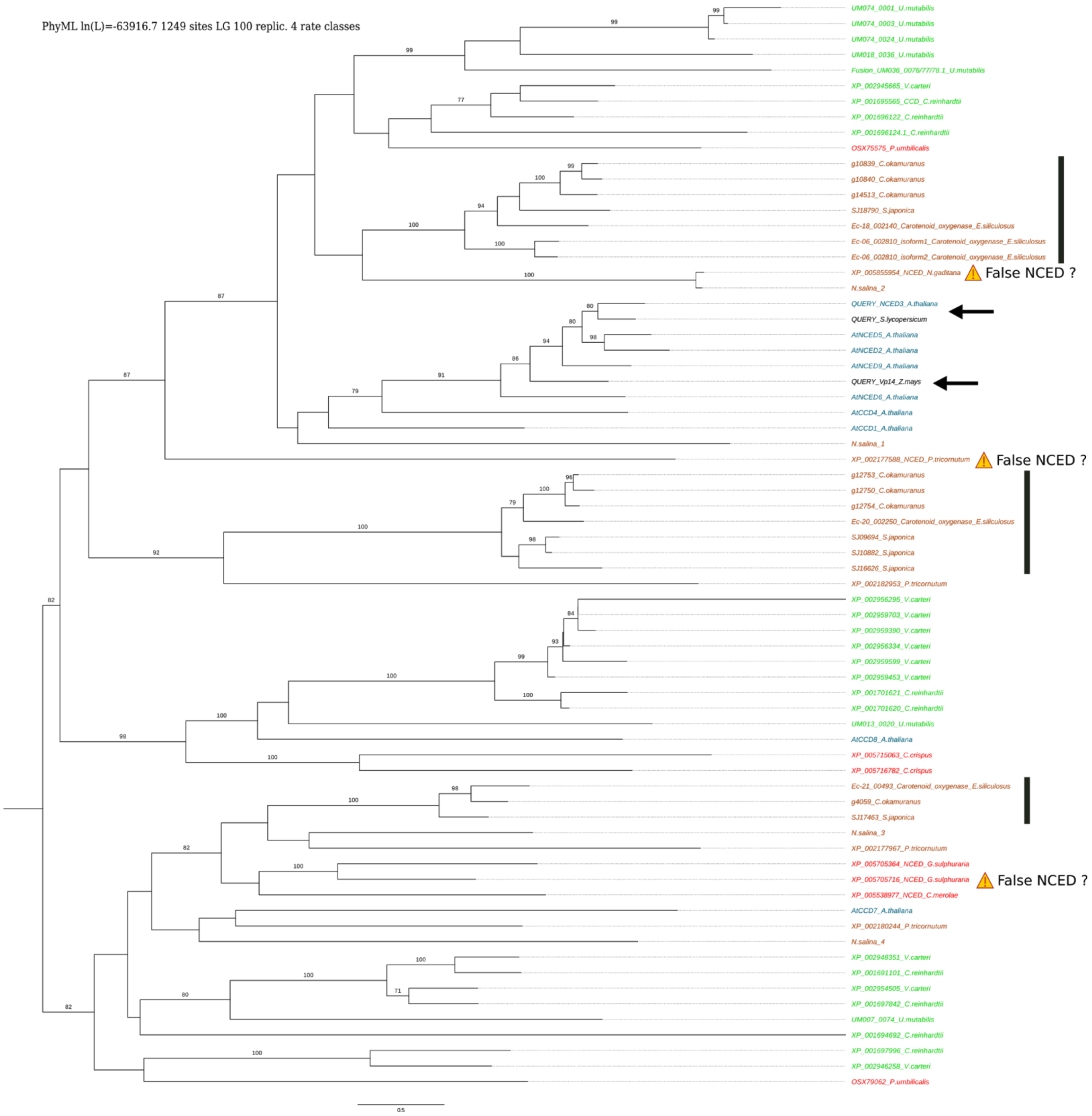

PhyML ln(L)=-63916.7 1249 sites LG 100 replic. 4 rate classes
UM074_0001_U.mutabilis
UM074_0003_U.mutabilis
UM074_0024_U.mutabilis
UM018_0036_U.mutabilis
Fusion_UM036_0076/77/78.1_U.mutabilis
XP_002945665_V.carteri
XP_001695565_CCD_C.reinhardtii
XP_001696122_C.reinhardtii
XP_001696124.1_C.reinhardtii
OSX75575_P.umbilicalis
g10839_C.okamuranus
g10840_C.okamuranus
g14513_C.okamuranus
SJ18790_S.japonica
Ec-18_002140_Carotenoid_oxygenase_E.siliculosus
Ec-06_002810_isoform1_Carotenoid_oxygenase_E.siliculosus
Ec-06_002810_isoform2_Carotenoid_oxygenase_E.siliculosus
XP_005855954_NCED_N.gaditana
False NCED ?
N.salina_2
QUERY_NCED3_A.thaliana
QUERY_S.lycopersicum
AtNCED5_A.thaliana
AtNCED2_A.thaliana
AtNCED9_A.thaliana
QUERY_Vp14_Z.mays
AtNCED6_A.thaliana
AtCCD4_A.thaliana
AtCCD1_A.thaliana
N.salina_1
XP_002177588_NCED_P.tricornutum
False NCED ?
g12753_C.okamuranus
g12750_C.okamuranus
g12754_C.okamuranus
Ec-20_002250_Carotenoid_oxygenase_E.siliculosus
SJ09694_S.japonica
SJ10882_S.japonica
SJ16626_S.japonica
XP_002182953_P.tricornutum
XP_002956295_V.carteri
XP_002959703_V.carteri
XP_002959390_V.carteri
XP_002956334_V.carteri
XP_002959599_V.carteri
XP_002959453_V.carteri
XP_001701621_C.reinhardtii
XP_001701620_C.reinhardtii
UM013_0020_U.mutabilis
AtCCD8_A.thaliana
XP_005715063_C.crispus
XP_005716782_C.crispus
Ec-21_00493_Carotenoid_oxygenase_E.siliculosus
g4059_C.okamuranus
SJ17463_S.japonica
N.salina_3
XP_002177967_P.tricornutum
XP_005705364_NCED_G.sulphuraria
XP_005705716_NCED_G.sulphuraria
False NCED ?
XP_005538977_NCED_C.merolae
AtCCD7_A.thaliana
XP_002180244_P.tricornutum
N.salina_4
XP_002948351_V.carteri
XP_001691101_C.reinhardtii
XP_002954505_V.carteri
XP_001697842_C.reinhardtii
UM007_0074_U.mutabilis
XP_001694692_C.reinhardtii
XP_001697996_C.reinhardtii
XP_002946258_V.carteri
OSX79062_P.umbilicalis
0.5

Figure S5: Maximum likelihood tree of the NCED family. All the sequences provided with an accession number came from the NCBI database. The sequences presented on the various trees were selected based either on homology searches with the sequences identified QUERY (black arrow) or based on publications. When available, the functional annotation of the sequences were added to the sequence names. Stramenopile sequences (brown algae and diatoms) are shown in brown, red algae sequences in red, green algae sequences in green and *Arabidopsis thaliana* sequences in blue. Only bootstrap values greater than or equal to 70 are displayed. Tree were rooted by midpoint.

The first oxidoreductase involved in ABA synthesis belongs to the carotenoid oxygenase family (CCO) and it is composed of a single protein domain: retinal pigment epithelial membrane protein (PF03055). In plants, this family is divided into two subfamilies, carotenoid cleavage dioxygenase (CCD) and the NCED [1–4]. There seems to be a common origin between *A. thaliana* CCDs and the various groups of algae, but the NCED family seems to have evolved only in embryophytes [5,6]. It would therefore appear that the sequences of *P. tricornutum*, *N. gaditana*, *G. sulphuraria* and *C. merolae* annotated NCED are incorrect. Nevertheless, homology search allows us to target a set of sequences belonging to the CCDs family. Phylogenetically, for each of the seaweeds, three groups of CCDs are detected and they could be involved in the synthesis of apocarotenoids such as retinal or carotenoid-derived volatiles [6]. The first is composed of a single sequence for *S. japonica* and three paralogues for *C. okamuranus*, the second of three paralogues per organism and the third include a single sequence per organism. For instance, in terrestrial plants, CCD1 is involved in the preliminary steps of beta-ionone synthesis whereas CCD7 and CCD8 are implicated in strigolactone synthesis [6,7].

References

1. Tan, B.-C.; Joseph, L.M.; Deng, W.-T.; Liu, L.; Li, Q.-B.; Cline, K.; McCarty, D.R. Molecular characterization of the *Arabidopsis* 9-cis epoxycarotenoid dioxygenase gene family. Plant J. 2003, 35, 44–56.
2. Sui, X.; Kiser, P.D.; von Lintig, J.; Palczewski, K. Structural basis of carotenoid cleavage: from bacteria to mammals. Arch. Biochem. Biophys. 2013, 539.
3. Walter, M.H.; Strack, D. Carotenoids and their cleavage products: Biosynthesis and functions. Nat. Prod. Rep. 2011, 28, 663.
4. Rodriguez-Concepcion, M.; Avalos, J.; Bonet, M.L.; Boronat, A.; Gomez-Gomez, L.; Hornero-Mendez, D.; Limon, M.C.; Meléndez-Martínez, A.J.; Olmedilla-Alonso, B.; Palou, A.; et al. A global perspective on carotenoids: Metabolism, biotechnology, and benefits for nutrition and health. Prog. Lipid Res. 2018, 70, 62–93.
5. Priya, R.; Siva, R. Phylogenetic analysis and evolutionary studies of plant carotenoid cleavage dioxygenase gene. Gene 2014, 548, 223–233.
6. Ahrazem, O.; Gómez-Gómez, L.; Rodrigo, M.J.; Avalos, J.; Limón, M.C. Carotenoid Cleavage Oxygenases from Microbes and Photosynthetic Organisms: Features and Functions. Int. J. Mol. Sci. 2016, 17.
7. Harrison, P.J.; Bugg, T.D.H. Enzymology of the carotenoid cleavage dioxygenases: Reaction mechanisms, inhibition and biochemical roles. Arch. Biochem. Biophys. 2014, 544, 105–111.

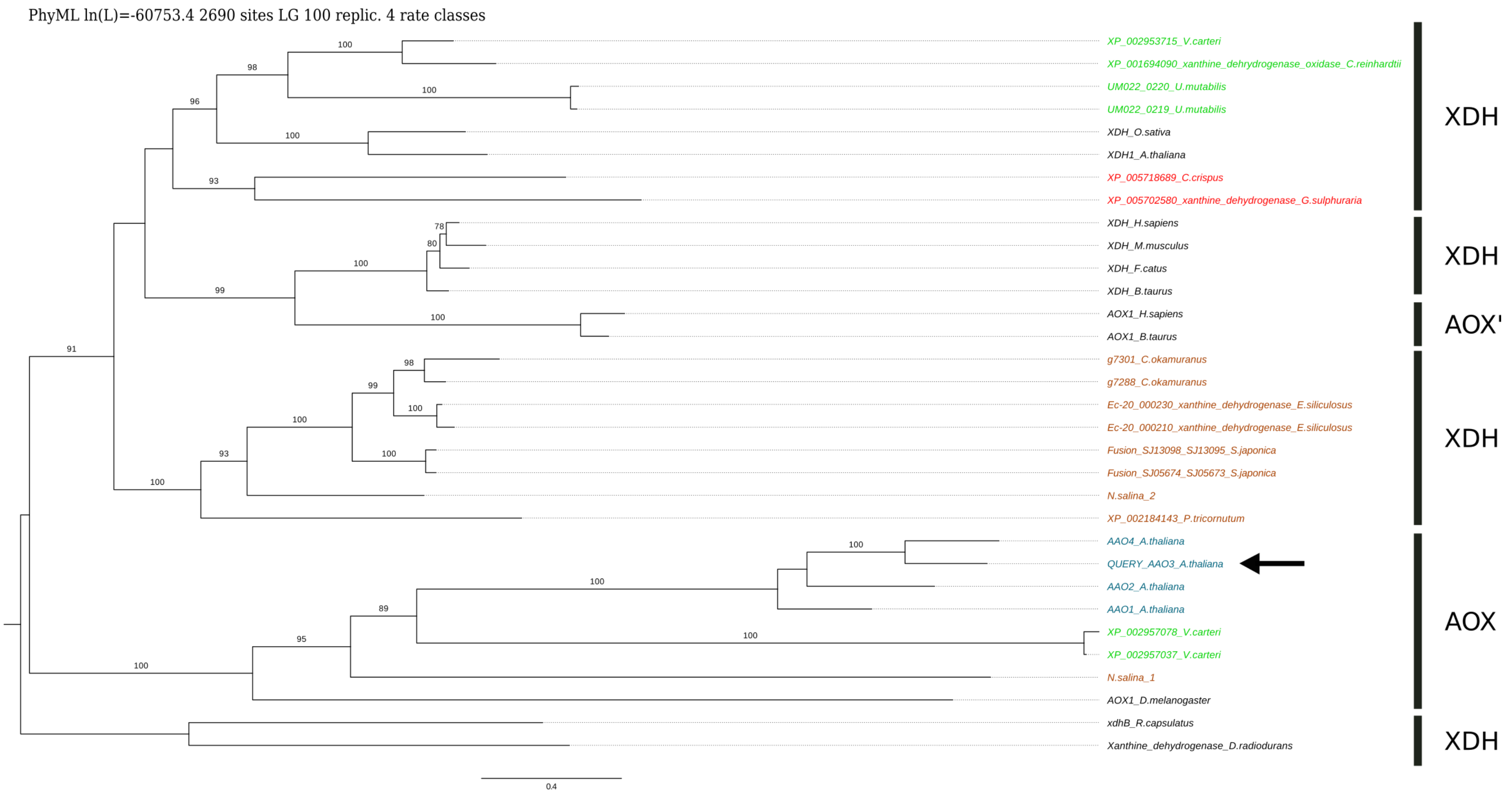

Figure S6: Maximum likelihood tree of the xanthine oxidase family. All the sequences provided with an accession number came from the NCBI database. The sequences presented on the various trees were selected based either on homology searches with the sequences identified QUERY (black arrow) or based on publications. When available, the functional annotation of the sequences were added to the sequence names. Stramenopile sequences (brown algae and diatoms) are shown in brown, red algae sequences in red, green algae sequences in green and *Arabidopsis thaliana* sequences in blue. Only bootstrap values greater than or equal to 70 are displayed. Tree were rooted by two bacterial sequences (*R. capsulatus* and *D. radiodurans*).

The third oxidoreductase involved in ABA synthesis belongs to the aldehyde oxygenase (AO) family [1]. AO is a subfamily of xanthine oxidase (XO) which also includes xanthine dehydrogenase (XDH). The AOs have evolved independently twice from XDH paralogues [2], which explains why these sequences have a similar structure and organization in protein domains (six domains in total: 2Fe-2S iron-sulfur cluster binding domain PF00111, [2Fe-2S] binding domain PF01799, FAD binding domain in molybdopterin dehydrogenase PF00941, CO dehydrogenase flavoprotein C-terminal domain PF03450, Aldehyde oxidase and xanthine dehydrogenase, a/b hammerhead domain PF01315, Molybdopterin-binding domain of aldehyde dehydrogenase PF02738). No AOs were detected in the brown and red algae analysed, suggesting a gene loss of in those species. On the other hand, the homology search, confirmed by the annotation files, allowed us to identify the presence of XDH (involved in the purine degradation pathways) in these two types of algae.

References

1. Seo, M.; Aoki, H.; Koiwai, H.; Kamiya, Y.; Nambara, E.; Koshiba, T. Comparative Studies on the *Arabidopsis* Aldehyde Oxidase (AAO) Gene Family Revealed a Major Role of AAO3 in ABA Biosynthesis in Seeds. *Plant Cell Physiol.* **2004**, *45*, 1694–1703.
2. Rodríguez-Trelles, F.; Tarrío, R.; Ayala, F.J. Convergent neofunctionalization by positive Darwinian selection after ancient recurrent duplications of the xanthine dehydrogenase gene. *Proc. Natl. Acad. Sci. U. S. A.* **2003**, *100*, 13413–13417.

	Saccharina japonica	*Cladosiphon okamuranus*
SDR5C	+	+
SDR12C		+
SDR17C	+	+
SDR19C		+
SDR35C	+	+
SDR65C	+	
SDR73C	+	+
SDR152C	+	
SDR172C	+	+
SDR335C		+
SDR380C	+	+
SDR574C	+	+
SDR87D	+	+
SDR350U		+

Table S9: Type of SDR found in the genomes of *S. japonica* and *C. okamuranus*. Identification based on http://www.sdr-enzymes.org. The second oxidoreductase involved in ABA synthesis belongs to the short-chain dehydrogenase (SDR) family and more specifically carries the Enoyl-(Acyl carrier protein) reductase domain (PF13561). This family includes a large number of subdivisions with various roles. ABA2 belongs to the SDR110C group of enzymes that perform six different functions [1]. Among the 14 types of SDR found in brown, none of them are related to the type sought. ABA2 is consequently an enzyme specific to terrestrial plants.

Among the 14 types of SDR: 8 types are shared between *S. japonica* and *C. okamuranus*, 2 are specific to *S. japonica* and 4 are specific to *C. okamuranus*. Within the algal genomes 52 (*S. japonica*) and 54 sequences (*C. okamuranus*) have been identified as potential SDRs, 10 and 13 of these sequences (~22%) could be annotated with an SDR number. For the other sequences only the type (essentially U or C) or otherwise the corresponding protein domain could be found (only concerns 2 sequences of *C. okamuranus*). According to homology research carried out within the 13 algae proteomes, it would appear that SDR17C and SDR152C are shared between green, red and brown algae, SDR65C between certain brown and green algae and that SDR87D is only found in *Cladosiphon okamuranus* and *Ectocarpus siliculosus* proteomes.

Reference

1. Moummou, H.; Kallberg, Y.; Tonfack, L.B.; Persson, B.; van der Rest, B. The Plant Short-Chain Dehydrogenase (SDR) superfamily: genome-wide inventory and diversification patterns. BMC Plant Biol. 2012, 12, 219.

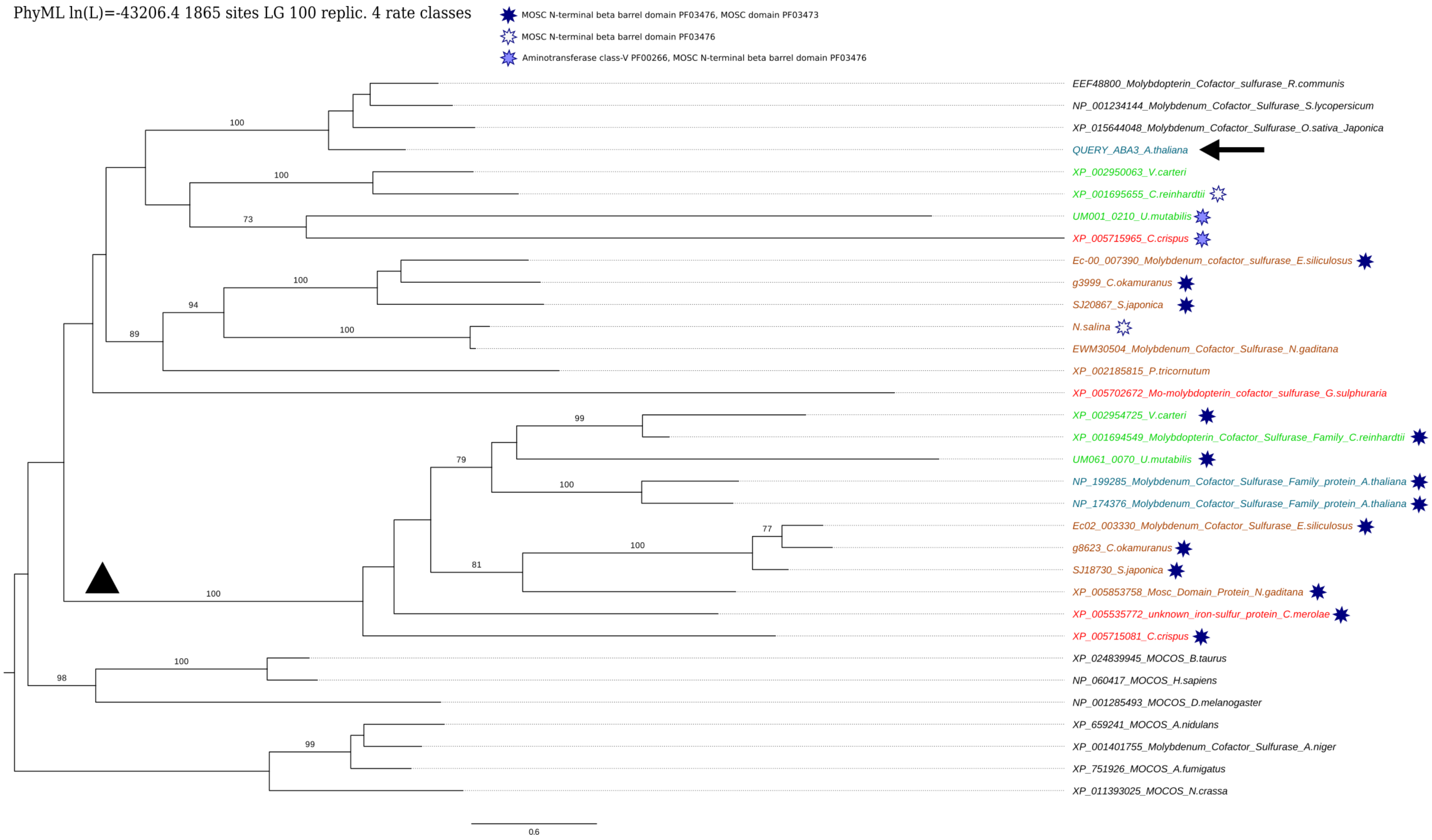

Figure S7: Maximum likelihood tree of the Mocos family. All the sequences provided with an accession number came from the NCBI database. The sequences presented on the various trees were selected based either on homology searches with the sequences identified QUERY (black arrow) or based on publications. When available, the functional annotation of the sequences were added to the sequence names. Stramenopile sequences (brown algae and diatoms) are shown in brown, red algae sequences in red, green algae sequences in green and *Arabidopsis thaliana* sequences in blue. Only bootstrap values greater than or equal to 70 are displayed. Tree were rooted with a set of Fungi sequences.

AAO3 requires the use of a molybdenum cofactor, ABA3 for its activation through the transfer of a sulphate group [1]. This type of enzyme is essential to activate both AO and XDH [2–6]. These enzymes are generally composed of three domains (Aminotransferase class-V PF00266, MOSC N-terminal beta barrel domain PF03476, MOSC domain PF03473). When the sequences in the tree are not composed of these three domains, a star is displayed next to their name. The black triangle corresponds to the probable loss of the domain Aminotransferase class-V PF00266. Finally, homology search seems to reveal two types of Mocos within the predicted protein sequences from brown algae.

References

1. Bittner, F.; Oreb, M.; Mendel, R.R. ABA3 Is a Molybdenum Cofactor Sulfurase Required for Activation of Aldehyde Oxidase and Xanthine Dehydrogenase in *Arabidopsis thaliana*. *J. Biol. Chem.* **2001**, *276*, 40381–40384.
2. Peng, T.; Xu, Y.; Zhang, Y. Comparative genomics of molybdenum utilization in prokaryotes and eukaryotes. *BMC Genomics* **2018**, *19*.
3. Filiz, E.; Distelfeld, A.; Fahima, T.; Karakas Metin, O.; Nevo, E.; Weining, S.; Altınkut Uncuoğlu, A. Barley molybdenum cofactor sulfurase (MCSU): Sequencing, modeling, and its comparison to other higher plants. *Turk. J. Agric. For.* **2015**, *39*.
4. Mendel, R.R. The Molybdenum Cofactor. *J. Biol. Chem.* **2013**, *288*, 13165–13172.
5. Hille, R.; Nishino, T.; Bittner, F. Molybdenum enzymes in higher organisms. *Coord. Chem. Rev.* **2011**, *255*, 1179–1205.
6. Mendel, R.R.; Hänsch, R. Molybdoenzymes and molybdenum cofactor in plants. *J. Exp. Bot.* **2002**, *53*, 1689–1698.

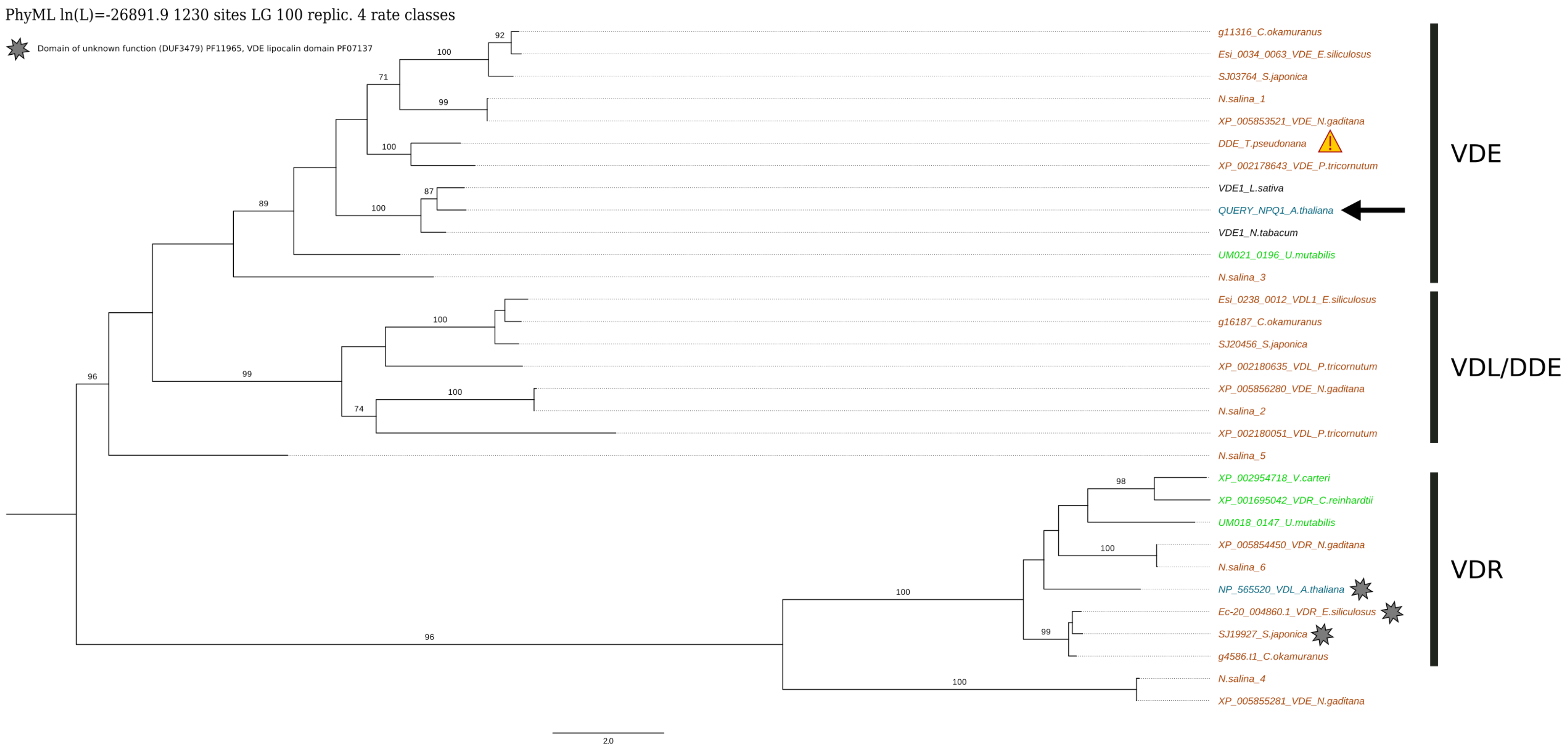

Figure S8: Maximum likelihood tree of the VDE. All the sequences provided with an accession number came from the NCBI database. The sequences presented on the various trees were selected based either on homology searches with the sequences identified QUERY (black arrow) or based on publications. When available, the functional annotation of the sequences were added to the sequence names. Stramenopile sequences (brown algae, *N. salina* and diatoms) are shown in brown, green algae sequences are in green and *Arabidopsis thaliana* sequences are in blue. Only bootstrap values greater than or equal to 70 are displayed. Tree was rooted by midpoint.

VDE is one of the two enzymes involved in the first xanthophyll cycle. It is composed of a single domain: VDE lipocalin domain (PF07137). As expected, three out-paralogs of VDE are found in brown algae proteomes, no copies of VDE gene are found in the red algae proteomes and the presence of a VDL appears to be a specificity of Phaeophyceae. The results obtained are consistent with those presented in **[1]** and **[2]**. The three sequences (*A. thaliana* even if it is annotated VDL, *E. siliculosus* and *S. japonica*) marked with a star and identified as VDR have two domains: domain of unknown function DUF3479 (PF11965) and VDE lipocalin domain (PF07137). The second cycle of xanthophyll, specific of Stramenopiles, involves a similar enzyme called DDE, which could correspond to VDL **[2–4]**. This enzyme is currently not biochemically characterized. Indeed, the only available DDE-labelled sequence (*T. pseudonana*) is phylogenetically close to VDE and not to VDL.

References

1. Coesel, S.; Oborník, M.; Varela, J.; Falciatore, A.; Bowler, C. Evolutionary Origins and Functions of the Carotenoid Biosynthetic Pathway in Marine Diatoms. *PLoS ONE* 2008, *3*.
2. Mikami, K.; Hosokawa, M. Biosynthetic Pathway and Health Benefits of Fucoxanthin, an Algae-Specific Xanthophyll in Brown Seaweeds. *Int. J. Mol. Sci.* 2013, *14*, 13763–13781.
3. Kuczynska, P.; Jemiola-Rzeminska, M.; Strzalka, K. Photosynthetic Pigments in Diatoms. *Mar. Drugs* 2015, *13*, 5847–5881.
4. Dambek, M.; Eilers, U.; Breitenbach, J.; Steiger, S.; Büchel, C.; Sandmann, G. Biosynthesis of fucoxanthin and diadinoxanthin and function of initial pathway genes in *Phaeodactylum tricornutum*. *J. Exp. Bot.* 2012, *63*, 5607–5612.

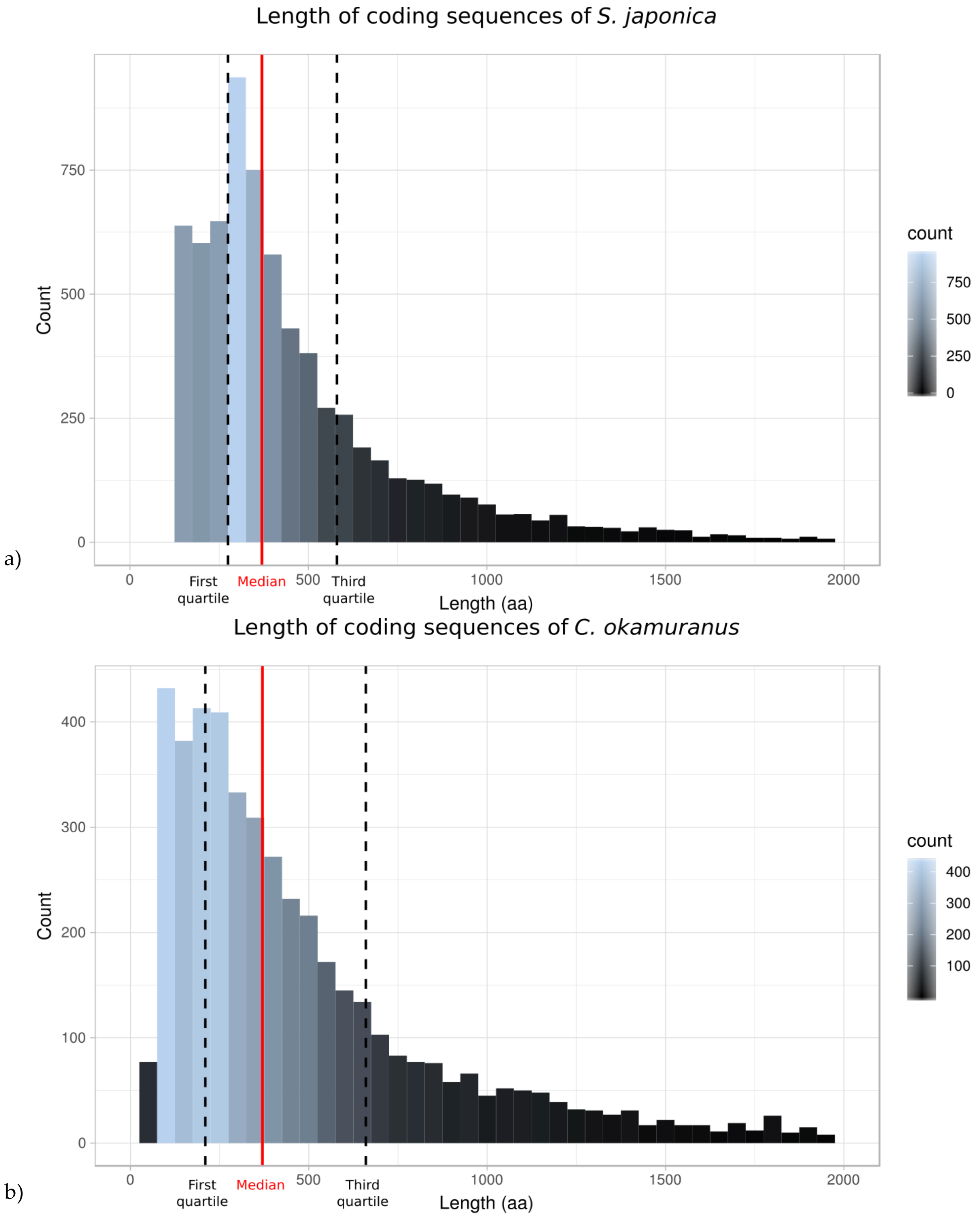

Figure S9: Amino acid length of unannotated coding sequences of *S. japonica* (a) and *C. okamuranus* (b). Median (red line) and quartiles (black dot lines) are shown. Range of 50 amino acids is display. **a.1**) The average size of the 7,072 unannotated sequences of *S. japonica* is 506 amino acids and the median is at 373. Measuring intervals: [130 (min) - 268];] 268 – 373],] 373 - 506];] 506 - 13,869 (max)] **a.2)** The average size of the 4,655 unannotated sequences of *C. okamuranus* is 550 amino acids and the median is at 371. Measuring intervals: [11 (min) - 208];] 208 – 371],] 371 - 662];] 662 - 6,638 (max)].

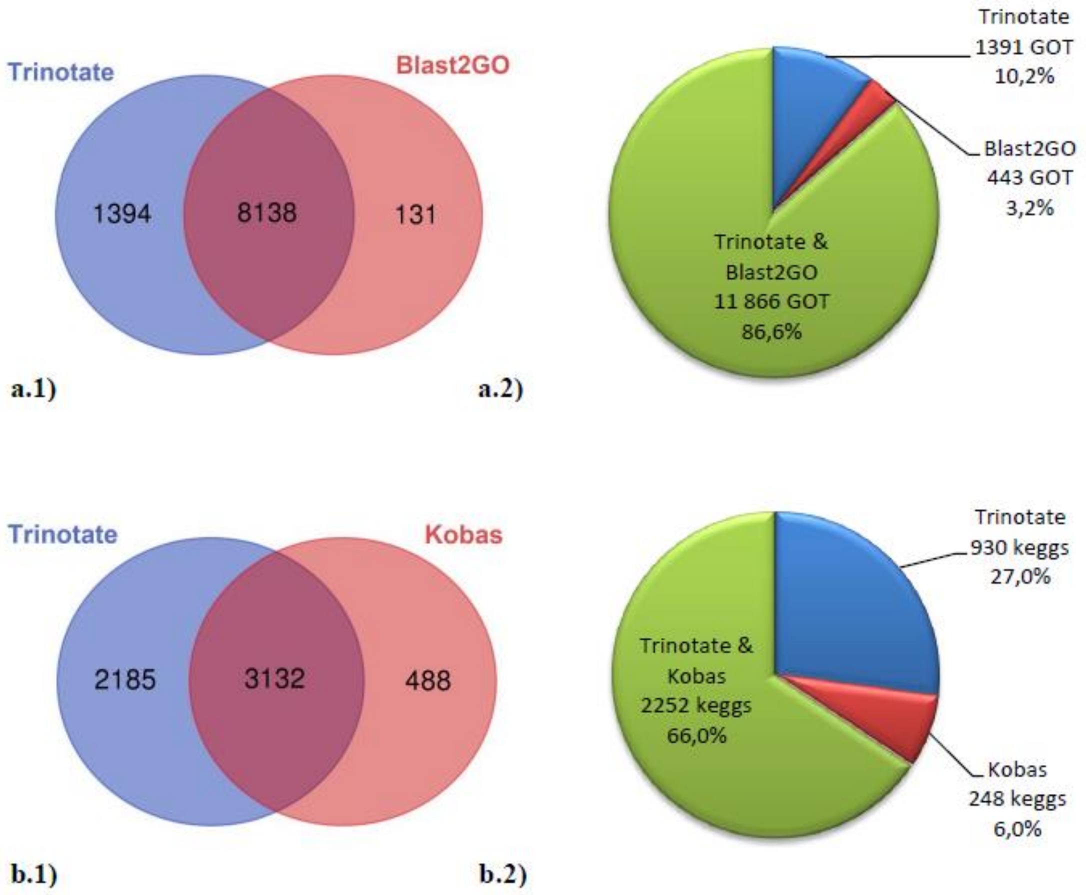

Figure S10: Enrichment of the annotation in GO terms and KEGG identifiers: data fusion. **a.1**) Of the 9,663 sequences annotated by one or more GO terms, 14.4% and 1.4% come exclusively and respectively from the results of Trinotate and Blast2GO. **a.2)** At this stage the Gene ontology is composed of 47,176 different terms (go_basic.obo version 1.2 - date 04/30/2018). After the enrichment with Blast2GO, the annotation of the *S. japonica* genome involves 13,701 different GO terms, or 29.0% of the Gene Ontology. Of these 13,701 unique GO terms, 10.2% and 3.2% were derived respectively from the results of Trinotate and Blast2GO. **b.1)** Of the 5,805 sequences annotated by at least one KEGG identifier, 37.6% and 8.4% were derived exclusively and respectively from the results of Trinotate and Kobas. **b.2)** Once the information from Trinotate and Kobas has been merged, there are 3,430 unique KEGG identifiers, 27% and 6% come respectively from the results of Trinotate and Kobas.
